# Observability of Paramagnetic NMR Signals at over 10 000 ppm Chemical Shifts

**DOI:** 10.1002/anie.202107944

**Published:** 2021-09-15

**Authors:** Jonas C. Ott, Elizaveta A. Suturina, Ilya Kuprov, Joscha Nehrkorn, Alexander Schnegg, Markus Enders, Lutz H. Gade

**Affiliations:** ^1^ Anorganisch-Chemisches Institut Universität Heidelberg Im Neuenheimer Feld 276 69120 Heidelberg Germany; ^2^ Department of Chemistry University of Bath Bath BA2 7AY UK; ^3^ School of Chemistry University of Southampton Southampton SO17 1BJ UK; ^4^ EPR Research Group MPI for Chemical Energy Conversion Stiftstrasse 34–36 45470 Mülheim Ruhr Germany

**Keywords:** iron, paramagnetic NMR, relaxation, THz-EPR, zero-field splitting

## Abstract

We report an experimental observation of ^31^P NMR resonances shifted by over 10 000 ppm (meaning percent range, and a new record for solutions), and similar ^1^H chemical shifts, in an intermediate‐spin square planar ferrous complex [^*t*Bu^(PNP)Fe‐H], where PNP is a carbazole‐based pincer ligand. Using a combination of electronic structure theory, nuclear magnetic resonance, magnetometry, and terahertz electron paramagnetic resonance, the influence of magnetic anisotropy and zero‐field splitting on the paramagnetic shift and relaxation enhancement is investigated. Detailed spin dynamics simulations indicate that, even with relatively slow electron spin relaxation (*T*_1_ ≈10^−11^ s), it remains possible to observe NMR signals of directly metal‐bonded atoms because pronounced rhombicity in the electron zero‐field splitting reduces nuclear paramagnetic relaxation enhancement.

## Graphical abstract

High‐resolution paramagnetic NMR resonances of a ferrous complex with an intermediate spin ground state with chemical shifts beyond ten thousand ppm were detected, the observability of which are attributed to a large rhombic zero‐field splitting resulting in an effective suppression of the dipolar relaxation mechanism.

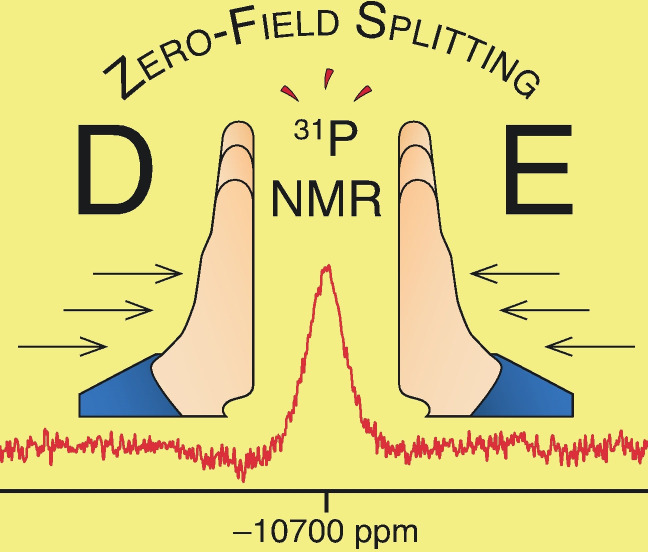

## Introduction

Nuclear magnetic resonance (NMR) spectroscopy of paramagnetic systems is complicated by the wide chemical shift range and rapid relaxation that broadens signals beyond detection.[^1^, ^2^] Assignment of NMR spectra and characterisation of open electron shell systems by this method remains challenging. In molecules with more than one unpaired electron, the theoretical treatment is significantly more complex than for mono‐radicals in organic or transition metal compounds.[^3^, ^4^] At the same time, there is a growing interest in the detection and analysis of paramagnetic intermediates in catalytic reaction cycles, particularly for the first‐row transition metals, where the reactive molecular fragments tend to be either directly bonded, or otherwise in close proximity to the metal.[^5^, ^6^, ^7^, ^8^, ^9^, ^10^, ^11^] Other areas of current interest are single‐molecule magnets (SMMs),[^12^, ^13^, ^14^, ^15^] contrast agents in magnetic resonance imaging (MRI),[^16^, ^17^, ^18^] and pseudocontact shift (PCS) probes in structural biology.[^19^, ^20^, ^21^, ^22^] Several recent studies used magneto‐structural correlations derived by multireference *ab initio* methods to refine chemical structures using paramagnetic NMR shifts.[^23^, ^24^, ^25^]

Magnetic properties of open‐shell compounds are frequently analysed by a combination of electron paramagnetic resonance (EPR) methods and superconducting quantum interference device (SQUID) magnetometry.[^26^, ^27^] However, direct measurement of zero‐field splitting (ZFS) and other electron spin Hamiltonian (SH) parameters in such systems is challenging, particularly in integer spin compounds. All sources of information are therefore valuable, and there is a growing interest in using paramagnetic NMR data to constrain electron SH parameters.[^28^, ^29^, ^30^, ^31^] The use of electronic structure theory in the analysis of paramagnetic NMR data remains a growing field of research, and the clarification of its fundamental aspects remains a focus of current investigations.[^32^, ^33^, ^34^, ^35^, ^36^, ^37^, ^38^, ^39^, ^40^, ^41^, ^42^]

Paramagnetic relaxation enhancement (PRE) is a useful source of information about electron‐nucleus distances and also provides estimates of electron relaxation rates.^[43]^ Currently, the analysis of PRE is mostly based on complementary models derived by Solomon, Bloembergen, and Morgan (contact and dipolar mechanism, referred to here as SBM mechanism),[^44^, ^45^, ^46^] and Guéron (Curie mechanism).^[47]^ Both approaches, in their original formulation, account only for the effective magnetic moment *μ*_eff_; the effects of ZFS and **g**‐tensor anisotropy are ignored. In a recent systematic study of non‐Gd lanthanide complexes the limitations of this approach were demonstrated.^[48]^ Although more elaborate theoretical models do exist,[^49^, ^50^, ^51^, ^52^, ^53^, ^54^, ^55^, ^56^, ^57^, ^58^, ^59^] they are exceedingly difficult to apply and are thus rarely used.

**Figure 1:**
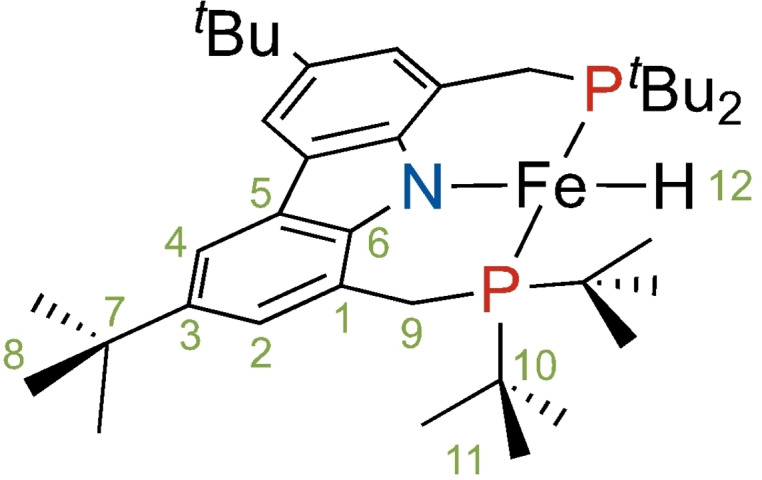
Intermediate‐spin ferrous hydrido complex [^*t*Bu^(PNP)Fe‐H] (**1**).

Our interest in this topic was sparked by the remarkable observation^[60]^ of the NMR signal of a directly metal‐bonded hydrogen atom in a square planar *S*=1 iron complex [^*t*Bu^(PNP)Fe‐H] shown in Figure 1 which, by the conventional wisdom, was not supposed to be detectable.[^2^, ^56^] As shown in this study, the attempt to observe the ^31^P NMR signal was equally successful, and yielded what appears to be the current record for ^31^P chemical shifts in liquid state NMR.

This study explores the electronic and magnetic properties that enable the observability of NMR signals of nuclei directly bound to a paramagnetic metal centre. A detailed investigation — using a combination of NMR, frequency‐domain Fourier transform (FD‐FT) THz‐EPR,[^61^, ^62^] magnetometry, and quantum chemistry — reveals the crucial role of zero‐field splitting and magnetic anisotropy in the mechanisms of nuclear spin relaxation. In our relaxation analysis, we employ a powerful but easily implemented method based on adiabatic elimination. Making reasonable assumptions about electron spin dynamics, it allows us to account for the effects of zero‐field splitting and **g**‐tensor anisotropy at any magnetic field.

## Results and Discussion

### Observation and qualitative analysis

**Figure 2:**
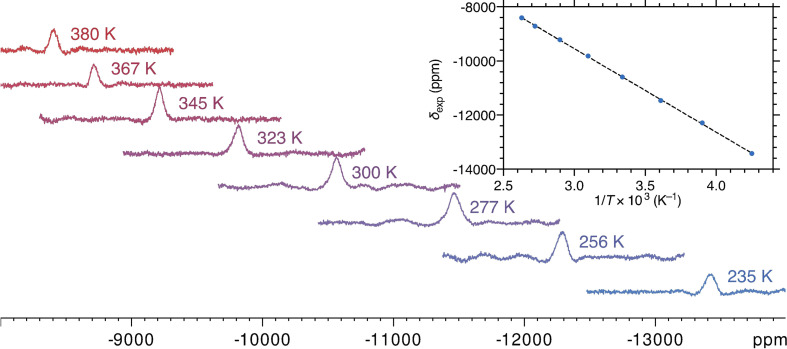
^31^P NMR spectra (4.7 Tesla, [D_8_]toluene) of complex **1** between 235 and 380 K, and the Curie plot of ^31^P chemical shift as a function of the inverse temperature. The dashed black line is a linear fit (slope −3.09×10^6^ ppm K, intercept −287.0 ppm, R^2^ 0.9998).

Following the experimental observation^[60]^ of ^1^H and ^13^C NMR signals in [^*t*Bu^(PNP)Fe‐H], our DFT estimates, augmented by contact and pseudocontact terms, predicted a ^31^P chemical shift of about −11 000 ppm at room temperature [B3LYP/6–311G(d,p); def2‐TZVP (Fe only)], close to where the signal was found experimentally (Figure 2) using 200, 400, and 600 MHz NMR spectrometers. In a 14.1 Tesla magnet, the ^31^P NMR resonance was broad and barely visible. However, it was readily observed at lower magnetic fields, with a line width of ≈5 000 Hz at 4.7 Tesla in a 200 MHz magnet. The chemical shift was found to display a Curie‐type temperature dependence (Figure 2, insert) between 235 and 380 K. The observation of ^31^P NMR resonances beyond −10 000 ppm (≡1 percent!) sets a new record in solution NMR spectroscopy. The first insight into this combination of extreme chemical shift and reasonably narrow NMR line is provided by *ab initio* ligand field theory (Supporting Information).

**Figure 3:**
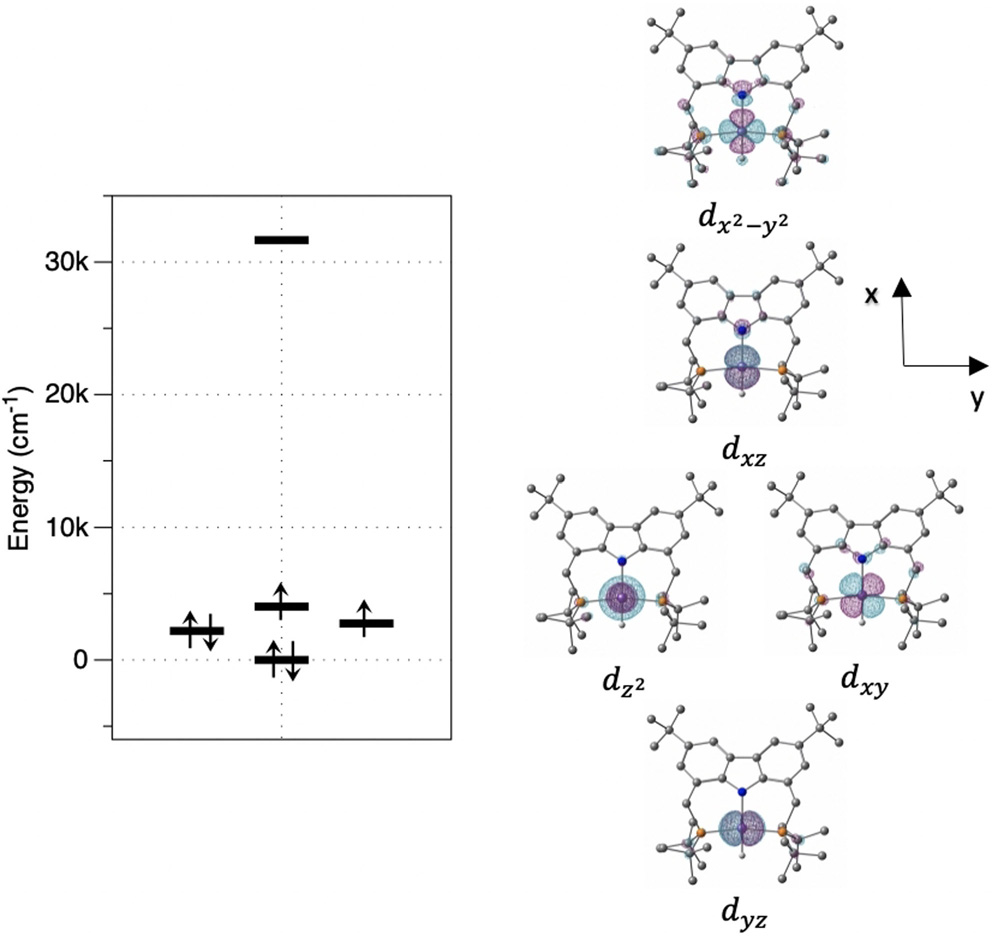
*Ab initio* ligand field theory computed splitting of the *d*‐orbital energies in the ligand field of the PNP pincer ligand and the hydrido ligand.

In Figure 3 the ligand field splitting of the *d*‐orbitals for the slightly distorted square‐planar complex **1** is displayed, in which the antibonding *d* ${}_{x{}^{2}-y{}^{2}}$ is at a considerably higher energy than the other *d*‐orbitals. Its non‐occupancy results in the observed intermediate‐spin ground state. This is significant, because the magnetic *d*_xz_ and *d*_xy_ orbitals have π‐symmetry and are close to antisymmetric with respect to 180‐degree rotation around Fe−H and Fe−P bonds, resulting in reduced direct spin delocalisation into the hydrogen 1s and phosphorus 3s orbitals, and thus reduced Fermi contact coupling.

### Analysis of Paramagnetic Chemical Shifts

The expression for the hyperfine shift tensor **δ**_HF_ of a nucleus becomes particularly elegant^[63]^ when the magnetic susceptibility tensor **χ** is expressed in the units of Å^3^ and the hyperfine coupling tensor **A** in units of ppm Å^−3^:

(1) $$\delta {}_{\mathrm{HF}}=\chi A$$

In isotropic solution state NMR, only the scalar part *δ*_HF_=$\frac{1}{3}$ Tr(**δ**_HF_) is observed. When relativistic corrections to the hyperfine coupling can be neglected, this part has two contributions:

(2) $$\delta {}_{\mathrm{HF}}=\delta {}_{\mathrm{FC}}+\delta {}_{\mathrm{PCS}}$$

where the Fermi contact (FC) part depends on the isotropic parts of the magnetic susceptibility and the hyperfine tensors

(3) $$\delta {}_{\mathrm{FC}}=\chi {}_{\mathrm{iso}}A{}_{\mathrm{iso}}$$

At room temperature, *χ*_iso_ is well approximated by the first term of the Taylor series^[64]^ with respect to 1/*T*, and *A*_iso_ is proportional to the spin density *ρ*_N_ at the nucleus:

(4) δFC=μ0μ2Bg2e(S+1)9kTρN

The pseudocontact component depends on the anisotropic part Δ**χ** of the susceptibility tensor and the dipolar part ***A***_dip_ of the hyperfine coupling tensor:

(5) $$\delta {}_{\mathrm{PCS}}=\frac{1}{3}\mathrm{Tr}\left(\Delta \chi \cdot A{}_{\mathrm{dip}}\right)$$

**Figure 4:**
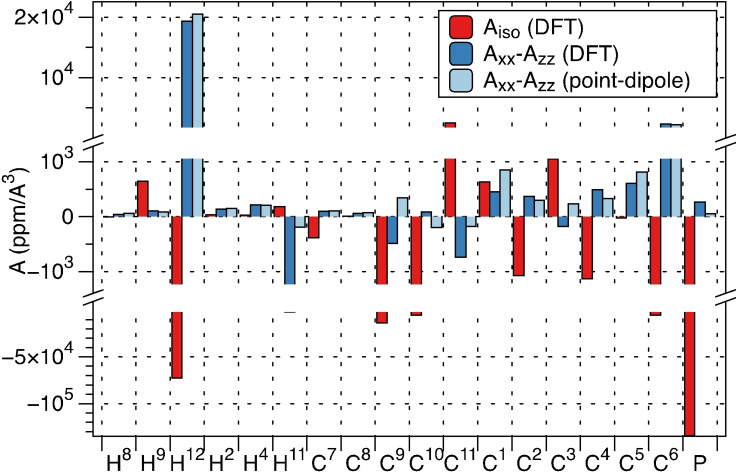
An illustration of the disagreement between the components of the hyperfine tensors computed with DFT (red for the isotropic part, dark blue for *A*_xx_−*A*_zz_) and those obtained from the point‐dipole approximation.

In the case at hand, we cannot use the popular point‐dipole approximation^[65]^ for **A**_dip_ because the extent of spin delocalisation relative to the electron‐nucleus distance is significant (Figure 4).

To analyse the paramagnetic shift we used the following special case of Eq. (1), which assumes mirror symmetry with respect to the XZ‐plane, where the X‐axis is along the Fe−H bond, and the Z‐axis is perpendicular to the H‐Fe‐P plane:

(6) $$\delta =\;\;\chi {}_{\mathrm{iso}}A{}_{\mathrm{iso}}+\frac{1}{3}\Delta \chi {}_{\mathrm{xx}}\left(A{}_{\mathrm{xx}}-A{}_{\mathrm{zz}}\right)+\frac{1}{3}\Delta \chi {}_{\mathrm{yy}}\left(A{}_{\mathrm{yy}}-A{}_{\mathrm{zz}}\right)+\frac{1}{3}\Delta \chi {}_{\mathrm{xz}}\left(A{}_{\mathrm{xz}}-A{}_{\mathrm{zx}}\right)$$

**Figure 5:**
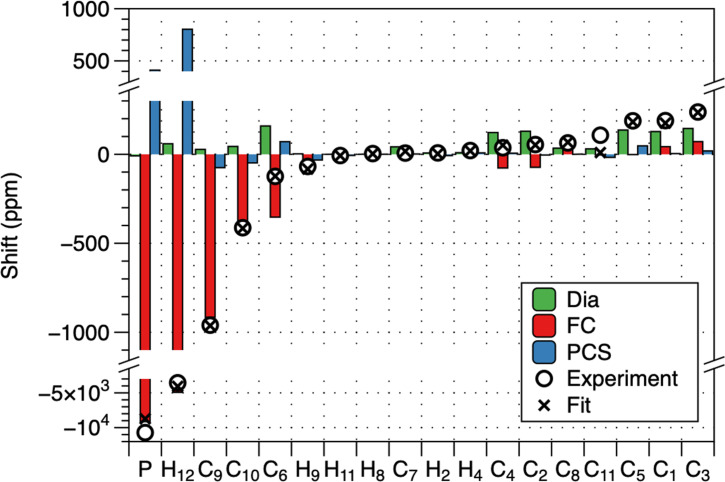
Contributions to the paramagnetic shifts at 295 K. The theoretical diamagnetic shift (M06‐L/6‐31G(d)) is shown in green, the Fermi contact contribution in red, the PCS is shown in blue (based on TPSSH/def2‐TZVP computed hyperfine tensors and best‐fit magnetic susceptibility tensor). The total calculated chemical shift (crosses) are compared with the experiment (circles).

The isotropic hyperfine component is very sensitive to the choice of the exchange‐correlation functional and basis set. In addition to that, a recent study has shown that DFT may overestimate the contact contribution.^[66]^ Five methods were tested (Supporting Information, Table S8); the best fit was achieved with TPSSH/def2‐TZVP. Signals strongly affected by rotational/conformational averaging (*t*Bu, Me, CH_2_) were excluded from the fit, as well as the signals of directly coordinated nuclei, as vibrational averaging might be needed due to highly non‐linear variation of hyperfine tensor even with small displacement along vibrational modes that affect the distance to the metal. The calculated contribution of the spin‐orbit coupling to the hyperfine tensor of H^12^ was 2 % for the isotropic part and ≈10 % for *A*_xx_−*A*_zz_. Both factors may be the reason for the small disagreement between calculated and experimental H^12^ shift (Figure 5). Calculated ^13^C and other ^1^H hyperfine tensors were largely unaffected by the spin‐orbit coupling effects, which were neglected in the subsequent analysis.

The paramagnetic shift (Figure 5) of all signals is dominated by Fermi contact contributions, however, the PCS is also significant as it contributes up to 50 % of the total shift for some signals. This has allowed us to constrain both isotropic and anisotropic components of the susceptibility tensor using the linear regression in Eq. (6); the Δ*χ*_xz_ component was found to be zero and was fixed at that value in further analysis. The latter may be due to the greater effective symmetry of the molecule on the NMR time scale in solution (*C*_2v_ instead of C_s_).

**Figure 6:**
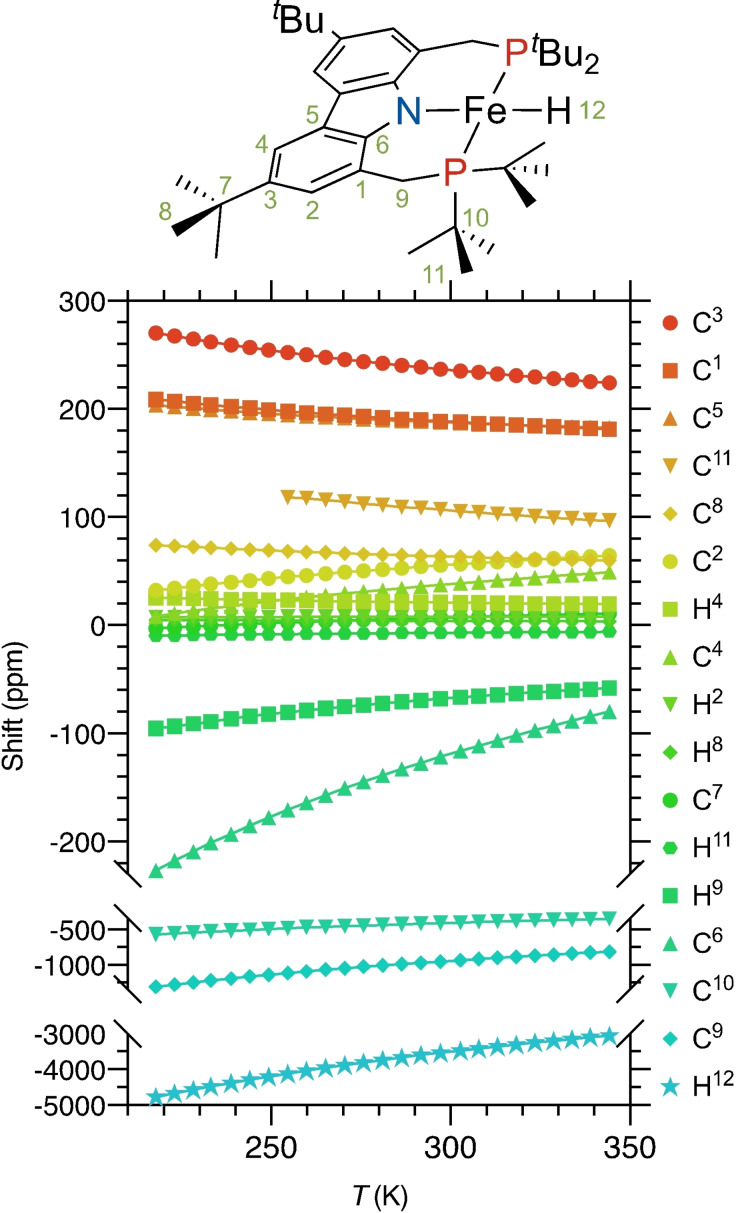
Temperature dependence of the observable ^1^H and ^13^C NMR resonances (14.1 Tesla, [D_8_]toluene). Solid lines represent the connecting lines between data points (symbols).

**Figure 7:**
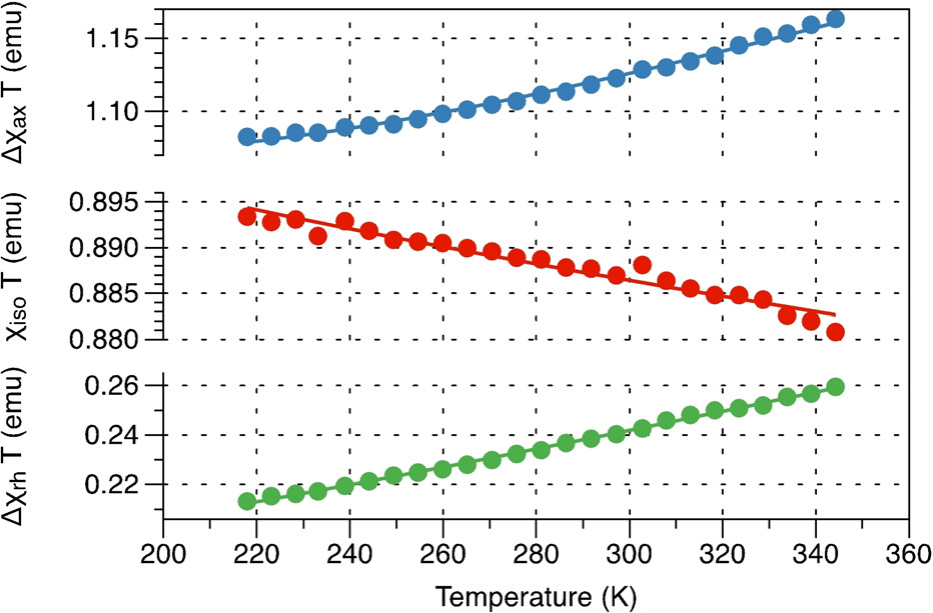
Temperature dependence of the isotropic part of the magnetic susceptibility, its axiality, and its rhombicity, extracted from paramagnetic chemical shift data.

The same approach was used to fit paramagnetic shifts at various temperatures in the range from 220 to 350 K (Figure 6) providing temperature dependence of the susceptibility tensor components (Figure 7).

Each component of the susceptibility tensor can be expressed as a function of the **g**‐tensor, ZFS parameters (*D* and *E*) and temperature.^[64]^ For *S*=1, the expression, written in terms of *D* and *E* of the second rank ZFS tensor, is:^[67]^

(7) β-1χij=2μ2BeβD+2coshβE×(e12β(D-E)sinh(12β(D+E))12β(D+E)gxigxj+e12β(D+E)sinh(12β(D-E))12β(D-E)gyigyj+sinh(βE)βEgzigzj)

where *β*=1/*k*_B_ *T* is used here to fit the temperature dependence of the isotropic and anisotropic components of the susceptibility tensor extracted from the paramagnetic chemical shifts (Figure 7).

The temperature dependence of the magnetic susceptibility was found to display a small decrease in the isotropic part and an increase in the anisotropy that could not be explained by the spin Hamiltonian model alone, and thus extra parameters accounting for anisotropic temperature‐independent contributions were required to fit the data. The fit is most sensitive to the **g**‐tensor anisotropy because, in the high‐temperature limit, **χ**‐tensor components depend only on the **g**‐tensor. ZFS parameters were fixed from FD‐FT THz‐EPR *D*=−54.5 cm^−1^ and *E*=−13.84 cm^−1^ (*vide infra*). The best fit **g**‐tensor eigenvalues are (1.748(3), 1.519(5), 2.289(2)) and the temperature‐independent correction is (0, −0.00087(1), 0.00073(1) emu). The temperature‐independent correction is unusual due to the negative sign and probably masks different effects appearing at higher temperatures.

### Analysis of SQUID Magnetometry and THz‐EPR

**Figure 8:**
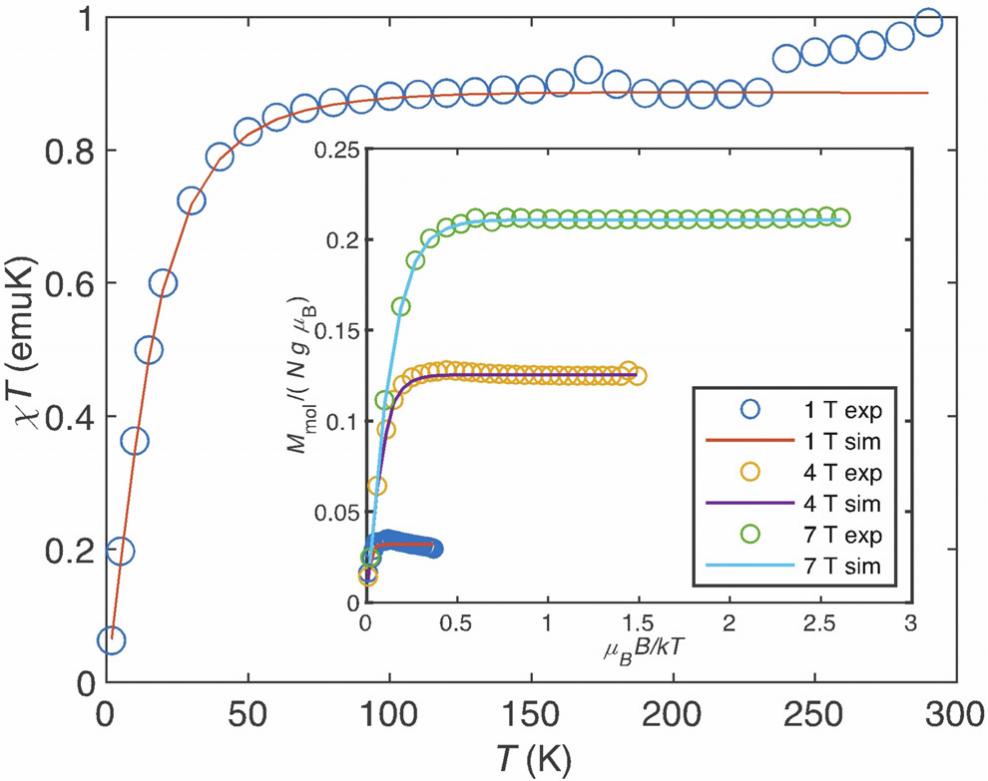
Magnetic susceptibility of complex **1** as *χT* product vs. temperature in toluene solution with a concentration of 9.67×10^−2^ mol/l. (inset) Experimental (circles) and simulated (solid lines) reduced magnetisation at 1 T, 4 T and 7 T of complex **1** in toluene solution. Simulations were obtained with *D*=−54 cm^−1^, |*E*|=14 cm^−1^, (|*E*/*D*|=0.26), *g*_x_=1.40, *g*_y_=1.90, *g*_z_=2.23 and a TIP of 0.00059 emu for the solution samples. The data obtained for a powder sample is presented in the Supporting Information.

Independent data related to the magnetic anisotropy were obtained from a combination of SQUID magnetometry and FD‐FT THz‐EPR spectroscopy. Figure 8 shows reduced magnetisation and magnetic susceptibility traces of **1** measured in toluene solution (cf. Supporting Information for data of neat powder) with *χT* values at room temperature of 1.1 and 0.9 cm^3^ K mol^−1^ for powder and solution, respectively. With *χT*≈$\frac{1}{8}$ *g* ^2^ *S*(*S*+1) and *S*=1, this corresponds to average *g*‐values close to 2.

Solution and powder traces display significant differences that may originate, *inter alia*, from inter‐molecular spin interactions in concentrated powders or uncertainties in the diamagnetic correction of the liquid solution data, and thus, magnetisation and susceptibility data of solution and powder samples could not be simulated with a unique set of electron SH parameters.

**Figure 9:**
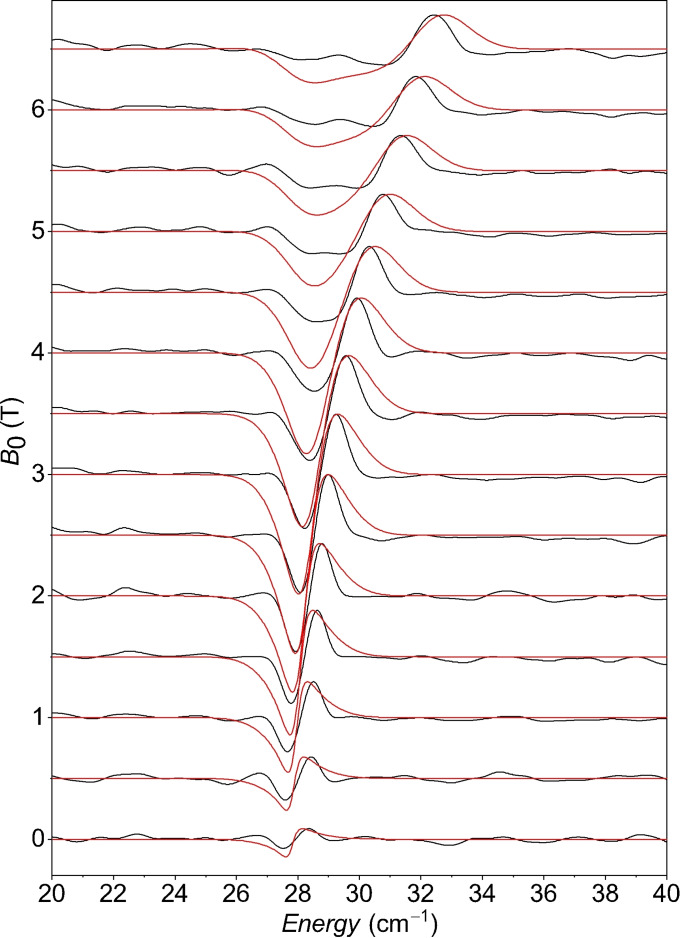
Experimental (black) and simulated (red) FD‐FT THz‐EPR magnetic division spectra (MDS) of pressed powder samples of **1** measured at *T=*5 K. MDS for different external magnetic fields *B*_0_ are obtained by dividing the raw spectrum measured at *B*_0_ by the spectrum measured at *B*_0_ + 1 T. Data is shown offset for *B*_0_ and rescaled with a global normalisation factor. In MDS, EPR resonances appear as negative (EPR resonance at *B*_0_) and positive (EPR resonance at *B*_0_ + 1 T) deviations from 1. Simulations with Eq. (8) were obtained with *D*=−54.5 cm^−1^, |*E*|=13.89 cm^−1^, (|*E*/*D*|=0.25), *g*_⊥_=1.65, *g*_∥_=2.63.

Better estimates of ZFS parameters were obtained from field‐dependent FD‐FT THz‐EPR, which allows for an accurate determination of large ZFS even in integer spin ‘EPR silent’ transition metal ions.[^68^, ^69^, ^70^] The instrument at BESSY II[^61^, ^62^] detects EPR in the frequency domain via FTIR transmission spectroscopy on samples that can be exposed to strong external magnetic fields and cryogenic temperatures; technical details are included in the Supplementary Information. Simulations were performed in EasySpin,[^71^, ^72^] assuming a pure *S*=1 spin Hamiltonian:

(8) H^=DS^2Z-13S^2+E(S^2X-S^2Y)+μBB0·g·S^

where the first two terms are axial and rhombic ZFS with axes labelled so that |*E*|≤|*D*|/3; the last term is the Zeeman interaction. Experimental THz‐EPR spectra and simulations are shown in Figure 9.

The simplest interpretation for the resonance at 27.5 cm^−1^ in an *S*=1 system would be axial ZFS with *D*=27.5 cm^−1^ and vanishing *E*. However, simulations with these parameters did not reproduce the field dependence of the FD‐FT THz‐EPR spectra (Supporting Information, Figure S5). The alternative scenario, where *D* is negative and the rhombicity in the ZFS lifts the degeneracy of the *m*_s_=±1 levels is well in accordance with the experimental data. This assumption predicts two EPR transitions at |*D*|+|*E*| and 2|*E*|. The observed transition at 27.5 cm^−1^ then corresponds to 2|*E*|≈27.5 cm^−1^. This yields |*E*|≈13.5 cm^−1^ and (due to |*E*|≤ |*D*|/3) creates a constraint on D < −41 cm^−1^. The second EPR transition is then expected at |*D*|+|*E*|, which would be above 54 cm^−1^ and therefore outside the spectral window of the data in Figure 9.

Further THz‐EPR measurements revealed magnetic field dependent peaks between 64 cm^−1^ and 70 cm^−1^ (Figure S5). We therefore chose the initial guess for *D* such that the second transition falls into the 64 cm^−1^ to 70 cm^−1^ range. Best match between simulation and experiment was found for *D*=−54.5 cm^−1^, |*E*|=13.89 cm^−1^, |*E*/*D*|=0.26, *g*_⊥_=1.65, *g*_∥_=2.63 (Figure 9). With this in place, we simulated the reduced magnetisation and *χT* data in Figure 8 with ZFS values as above and *g*‐values as fitting parameters. The overall agreement between simulations end experiments in Figures 8 and 9 supports the large negative ZFS.

Finally, a relativistic multireference SOC‐CASSCF(6,5)/ NEVPT2/def2‐TZVP calculation also predicted a large negative ZFS: the energy separation between the three magnetic sublevels of 27 cm^−1^ and 85 cm^−1^, and calculations using the effective Hamiltonian formalism yielded *D*=−71 cm^−1^ and a pronounced (|*E*/*D*|=0.191) rhombicity. The calculated **g**‐tensor eigenvalues (1.98, 2.06, 2.66) were also in reasonable agreement with the results obtained from SQUID and EPR data.

### Nuclear Relaxation Enhancement Analysis

The system studied in this work presents a difficult case of NMR spectroscopy of a nucleus so close to the unpaired electron that the point‐dipole approximation breaks down and the contact part of the hyperfine coupling is very large. We are also outside the Zeeman limit. In this situation, analytical expressions exist only for ZFS much stronger than the electron Zeeman splitting[^57^, ^58^, ^59^, ^73^] whereas intermediate magnetic fields are less researched, and mostly for hypothetical systems.[^50^, ^51^, ^52^, ^53^, ^55^, ^56^, ^74^, ^75^] Some of the expressions reported in the literature are not in practice computable. The situation therefore requires a radically different approach if any progress is to be made. We choose here to deal with the matter numerically; a detailed discussion of the prior art in paramagnetic relaxation enhancement may be found in the Supporting Information.

In the context of nuclear relaxation, we here advocate adiabatic elimination, which only makes one reasonable assumption: that the electron spin remains close to thermal equilibrium during the NMR experiment. We begin by partitioning the Liouville state space into the ‘slow’ subspace (nuclear states, state vector ***ρ***_0_) and the ‘fast’ subspace (electron states and electron‐nuclear correlations, state vector ***ρ***_1_). With that separation in place, the equation of motion acquires the following structure:

(9) ddtρ0ρ1=-iL00L01L10L11ρ0ρ1⇔{ρ˙0=-iL00ρ0-iL01ρ1ρ˙1=-iL10ρ0-iL11ρ1

where **L**_nk_ are the corresponding blocks of the Liouvillian. When we assume that the electron remains in the thermal equilibrium, this implies that ***ρ*** ˙_1_=0, and therefore (from the second equation in the system) that ***ρ***_1_=−L-111 **L**_10_ ***ρ***_0_. After placing this into the first equation, we obtain an effective equation of motion for the nuclear subspace:

(10) dρ0dt=-iL00ρ0+iL01L-111L10ρ0

where the second term on the right‐hand side is dissipative. This equation captures the long‐term evolution of nuclear spin states in the presence of a rapidly relaxing electron.

At the software implementation level, we partition the state space 𝔏 into the pure nuclear subspace 𝔑 (all states with the unit operator on the electron) and its complement 𝔏/𝔑 (all states involving electrons); the projector from 𝔏 into 𝔑 will be denoted **P**_𝔑_. The Liouvillian of the system at the molecular orientation *Ω*

(11) L(Ω)=[H(E)Z(Ω)+H(E)ZFS(Ω)+iR(E)(Ω)]⊗1(N)+HHFC(Ω)+1(E)⊗H(N)(Ω)

is generated using the standard *Spinach* ^5^ library functionality. Performing projections yields:
(12)

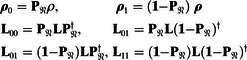




In our present case, electron dynamics is much faster than molecular rotation, and nuclear relaxation is much slower. Therefore, adiabatic elimination is valid at each orientation of the system, and the relaxation superoperator seen by the nucleus is an average:

(13) R(N)=i8π2∫L01(Ω)L-111(Ω)L10(Ω)dΩ

which is computed using standard 3‐angle powder grids supplied with *Spinach*. Under the indicated timescale separation assumptions, this equation imposes no conditions on the relative magnitude of the Zeeman interaction and ZFS, and accounts for all electron, electron‐nuclear, and nuclear spin interactions, both isotropic and anisotropic.

**Figure 10:**
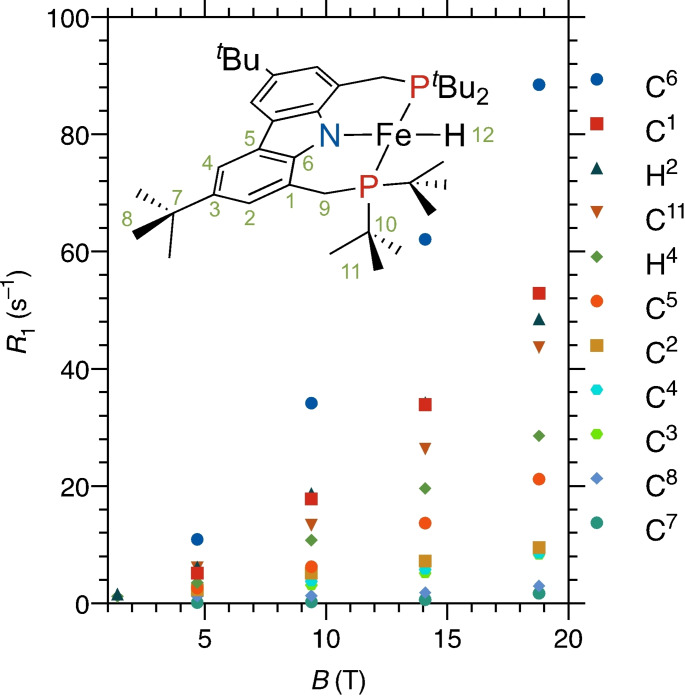
Plot of the longitudinal relaxation rates of the conformationally immobile nuclei of complex **1** against the applied magnetic field.

**Figure 11:**
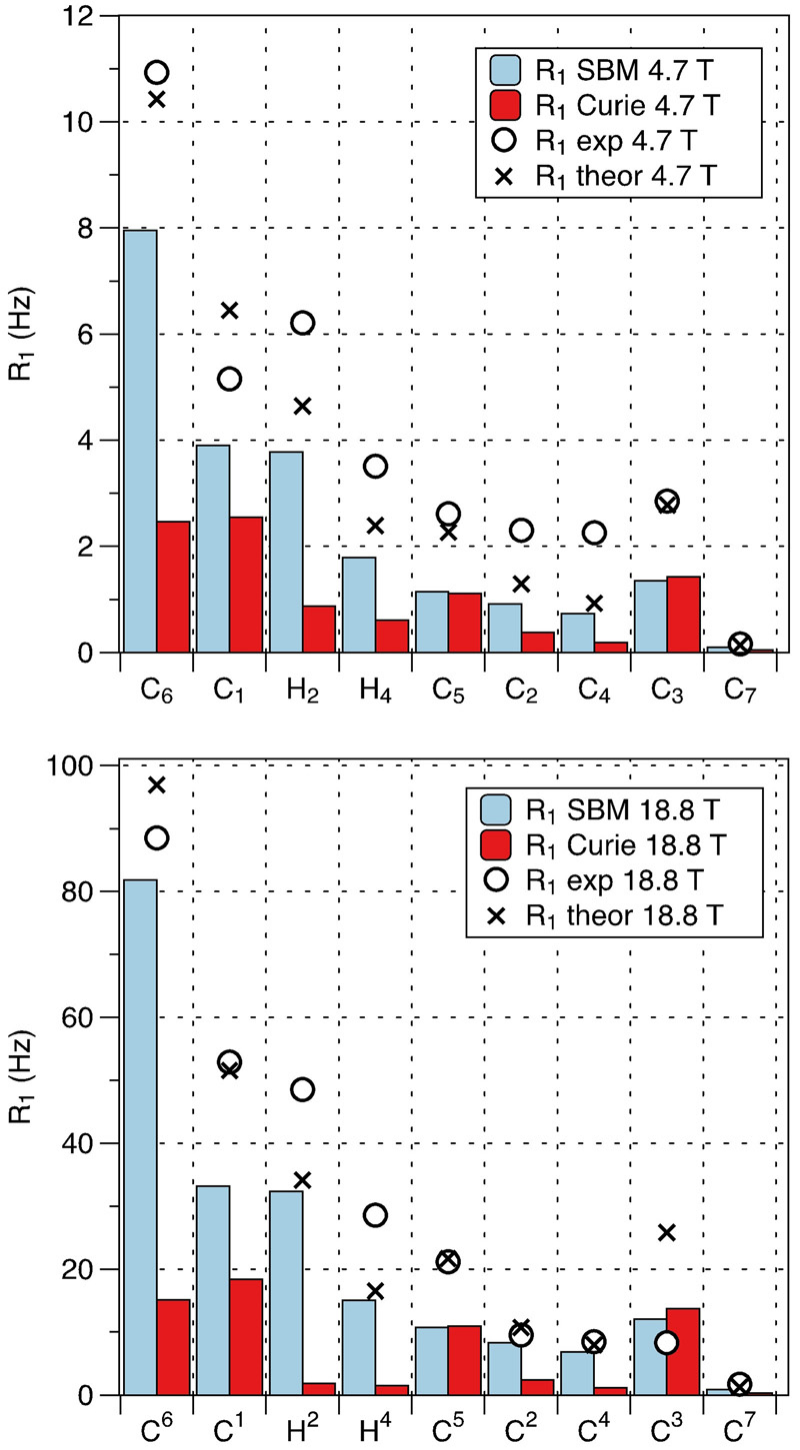
Experimental nuclear relaxation rates (*R*_1_ in Hz) measured at 4.7 T (top) and 18.8 T (bottom) shown in black circles, the best one‐parameter fit shown as crosses, the vertical bars show relative contributions of SBM (blue) and Curie (red) relaxation.

To determine the observability conditions for the nuclei directly bonded to the paramagnetic iron centre, an inspection of their relaxation behaviour is essential. Experimental measurements reveal a strong dependence of *R*_1N_ on the magnetic field (Figure 10). In the Zeeman limit, such behaviour would be attributable to Curie relaxation. However, an attempt to fit the data with just Curie relaxation that accounts for the anisotropic part of the susceptibility (as in the work by Fiat and Vega but with account for the anisotropy of the diamagnetic shielding; Supporting Information, Eq. (S6))[^48^, ^49^] was predictably unsuccessful — simulations of Curie relaxation using the magnetic susceptibility tensor derived from pNMR (*vide supra*), diamagnetic shielding, and hyperfine tensors calculated with DFT (Table S7) and rotational correlation time estimated with HYDRONMR^[76]^ (5.80×10^−10^ s) show that Curie mechanism covers only a fraction of the observed relaxation enhancement (Figure 11).

The ZFS parameters needed for the adiabatic elimination formalism were taken from the FD‐FT THz‐EPR data discussed above, the **g**‐tensor was determined from the variable temperature pNMR data, and hyperfine and diamagnetic shielding tensors modelled by DFT. One‐parameter fit of relaxation data for 10 different nuclei at 4 magnetic field strengths (Figure 10) yielded a fair agreement with experiment and a fitted electron relaxation rate *R*_e_=3.3(2) × 10^10^ s^−1^, which falls into the range expected for Fe^II^ complexes (Figure 11).^[2]^

**Figure 12:**
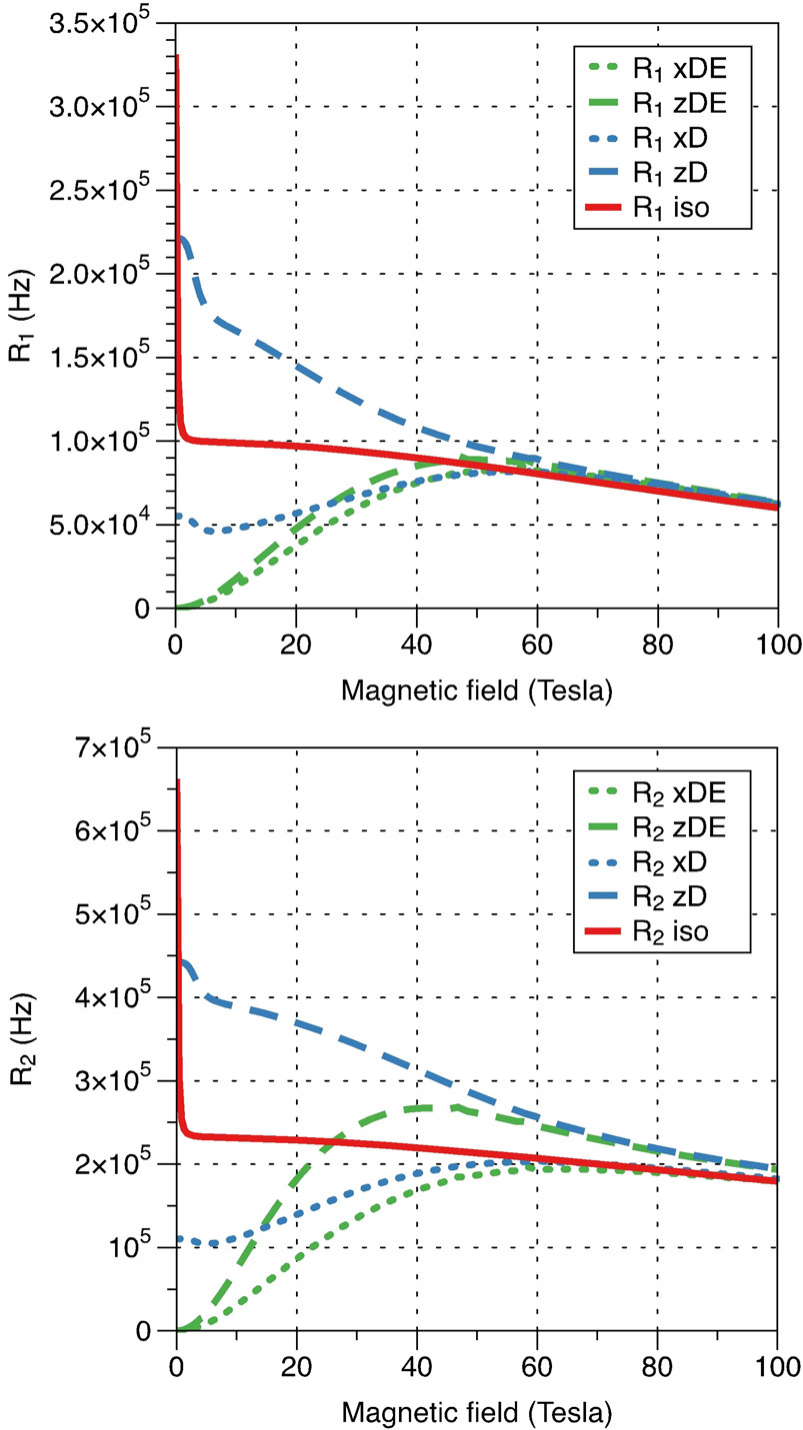
Simulated contact/dipolar relaxation rates (longitudinal — *R*_1_, top and transverse — *R*_2_, bottom) as a function of magnetic field. The model represents a point paramagnetic centre with *S*=1 and ^1^H located 1.57 Å away along either *x*‐axis (dotted lines) or *z*‐axis (dashed lines). The relaxation profile when no ZFS is present is shown in red, the case of axial ZFS with *D*=−56 cm^−1^ is shown in blue and rhombic ZFS with an additional *E*=−13.8 cm^−1^ is shown in green. The *g*‐tensor is assumed to be isotropic with *g*=2, and electron relaxation rate is 3.3×10^10^ s^−1^.

Simulations reveal the critical role of rhombic ZFS in the nuclear relaxation enhancement: non‐zero ZFS rhombicity slows down nuclear relaxation, particularly at low magnetic fields (Figure 12, green lines). Purely axial ZFS makes nuclear relaxation strongly direction dependent in the molecular frame of reference; it enhances the relaxation compared to the isotropic case along the main ZFS axis and reduces it in the perpendicular direction (Figure 12, blue lines).

With non‐zero ZFS rhombicity, SBM relaxation is negligible at very low field, but increases steeply with the magnetic field — the behaviour normally attributed to Curie relaxation.[^54^, ^58^] Similar behaviour is seen for longitudinal and transverse relaxation rates. Thus, the observability of the directly bonded ^1^H and ^31^P NMR signals in our intermediate‐spin Fe^II^ complex is mostly due to the large rhombic zero‐field splitting that suppresses the electron‐nuclear SBM relaxation mechanism; without it the NMR signals would be several orders of magnitude broader and thus impossible to detect.

## Conclusion

We have demonstrated how a combination of NMR, FD‐FT THz‐EPR, magnetometry, and quantum chemistry can reveal the significance of magnetic anisotropy for nuclear paramagnetic shift and paramagnetic relaxation enhancement. While ^1^H and ^13^C paramagnetic shifts are dominated by the contact contribution, the PCS component still remains significant in the iron complex [^*t*Bu^(PNP)Fe^II^‐H] (**1**) studied in this work. With the aid of computationally modelled diamagnetic shielding and hyperfine tensors we were able to extract the temperature dependence of isotropic and anisotropic parts of the susceptibility tensor from the positions of the NMR signals. We found that the anisotropic parts of the hyperfine tensors obtained with DFT are very different from the commonly used point‐dipole approximation, which must therefore be avoided in such systems.

The temperature dependence of the susceptibility tensor extracted from pNMR allowed us to estimate the **g**‐tensor anisotropy but not the ZFS. The precise value of *E* was obtained by FD‐FT THz‐EPR spectroscopy and the *D* value was confirmed with the help of SQUID magnetometry. Those parameters were used to fit nuclear relaxation rates and extract the electron relaxation rate, which was found to be *R*_e_=3.3(2) × 10^10^ s^−1^.

Nuclear relaxation due to the SBM mechanism is several orders of magnitude slower than would be expected from Solomon‐Bloembergen‐Morgan theory, which is therefore not applicable outside the Zeeman limit. A careful relaxation theory treatment using adiabatic elimination demonstrated that the large ZFS rhombicity in this system suppresses the SBM relaxation and therefore makes the NMR signals of ^1^H and ^31^P nuclei directly bonded to the paramagnetic metal centre observable. With this knowledge at hand, the NMR detectability of atoms directly bound to a paramagnetic metal centre is expected to be demonstrated for many more metal complexes in future studies, thus giving partial access to a previous ‘blind spot’ of the method.

## Conflict of interest

The authors declare no conflict of interest.

## Supporting information

As a service to our authors and readers, this journal provides supporting information supplied by the authors. Such materials are peer reviewed and may be re‐organized for online delivery, but are not copy‐edited or typeset. Technical support issues arising from supporting information (other than missing files) should be addressed to the authors.

Supporting Information
