# Supplementary material for: Observability of Paramagnetic NMR Signals at over 10 000 ppm Chemical Shifts
Source: Angew Chem Int Ed Engl. 2021 Sep 15;60(42):22856–64. doi: 10.1002/anie.202107944 (PMC8518043; doi:10.1002/anie.202107944)
Supplement: Supplementary file 1 — Supporting Information [file ANIE-60-22856-s001.pdf]

Angewandte Chemie
Eine Zeitschrift der Gesellschaft Deutscher Chemiker

Supporting Information

Observability of paramagnetic NMR signals at over 10 000 ppm chemical shifts

Jonas C. Ott, Elizaveta A. Suturina, Ilya Kuprov, Joscha Nehrkorn, Alexander Schnegg, Markus Enders,* and Lutz H. Gade**

anie_202107944_sm_miscellaneous_information.pdf

Supporting Information

S1 General Procedures 2
S2 NMR Spectroscopy 3
S3 FD-FT THz-EPR Spectroscopy 10
S4 SQUID Magnetometry 13
S5 NMR Shift Analysis 14
S6 Relaxation Theory 17
S7 Literature 20

S1 General Procedures

General Remarks

All experiments were carried out in oven-dried glassware and under argon atmosphere by using standard Schlenk or glovebox techniques. Argon 5.0 from Messer Group GmbH was used as inert gas and dried over Granusic phosphorous pentoxide before usage. All solvents were purchased anhydrous from Honeywell and either collected from a solvent purification system (M. Braun SPS 800) after being dried over activated alumina columns or dried and degassed by using standard methods and stored in glass ampules with Teflon plugs under an atmosphere of argon. Deuterated solvents were purchased from Sigma-Aldrich, dried and vacuum distilled accordingly and degassed by at least three successive freeze-pump-thaw cycles. Samples for NMR spectroscopy were prepared under argon in a glovebox, transferred to 5mm Wilmad precision NMR tubes attached to a Schlenk-system and sealed with a butane lighter torch under slightly reduced pressure. NMR spectra were recorded on Bruker spectrometers (vide infra). ^{1}H and ^{13}C NMR spectra were referenced to residual solvent resonances and are given in parts per million (ppm) relative to external tetramethylsilane. Complex [^{tBu}(PNP)FeH] (**1**) was synthesized according to literature.[1]

SQUID Magnetometry

Magnetic susceptibility of **1** was measured from powder samples of solid material immobilized in eicosane (powder) or in toluene solution (solution) in the temperature range 2 – 290 K by using a Superconducting Quantum Interference Device (SQUID) with a field of 1 T (MPMS-7, Quantum Design, calibrated with standard palladium reference sample, error < 2%). To correct for the diamagnetic contribution of the solvent and sample holders, reference measurements were carried with empty samples holders and samples holders filled with solvent. Multiple-field variable-temperature magnetization measurements were done at 1, 4, and 7 T in the temperature range 2 – 260 K with the magnetization equidistantly sampled on a 1/T temperature scale.

Computational Modelling

Geometry optimization was performed with B3LYP/6-311G(d,p) + def2-TZVP (Fe only) methods and the hyperfine coupling tensors was calculated with range of DFT methods and basis sets indicated in the text for C_s symmetrised geometry using Gaussian.[2] The diamagnetic shielding tensor was calculated with M06-L/6-31G(d)[3] for a structure where Fe was replaced with Zn and the diamagnetic shift was defined with respect to TMS reference. The magnetic susceptibility tensor was calculated with CASSCF(6,5)/NEVPT2 methods and def2-TZVP basis set with RI approximation where all 5 quintet, 45 triplet and 50 singlet states were included. These calculations and ab initio ligand field analysis were done with ORCA4.1.[4] Estimations of the spin-orbit effects on hyperfine were done with mean-field approximation and B3LYP/def2-TZVP basis set.

S2 NMR Spectroscopy

General Acquisition Procedure

Unless stated otherwise, the spectra were recorded at a temperature of 295 K. Bruker and Magritec spectrometers (Magritek Spinsolve 60, Bruker DRX200, Bruker AVII 400, Bruker AVIII 600, Bruker AVIII 800) were used for the data collection. All temperature dependent ^{1}H and ^{13}C NMR spectra were recorded on a Bruker AVIII 600 MHz spectrometer equipped with a BBO probe (room temperature probe). The temperature of the measurements was calibrated using methanol and glycol (80% in DMSO) as standard substances.[5] Relaxation times were determined by the inverse-recovery method. Due to a non-uniform excitation profile of the 180° pulse within the inversion recovery pulse sequence, the measurements had to be performed with excitation frequencies near the resonance of the individual signals. For the detection of the ^{31}P NMR signal the excitation frequency was set "on resonance". Moving the excitation frequency away from the resonance signal lead to disappearance of the signal. The identity of a real ^{31}P NMR signal was verified by following the NMR shift at different temperatures and by measuring NMR tubes containing the solvent only. The data acquisition parameters used for the paramagnetic ^{1}H, ^{13}C and ^{31}P NMR experiments are displayed in Tables S1-S3.

Table S1. Acquisition parameters for inverse-recovery ^{1}H and ^{13}C NMR experiments with different spectrometers.

Spectrometer	DRX 200		AVII 400		AVIII 600		AVIII 800	
Probe type	TBO		BBFO		BBO		CP-TCI	
Nucleus	^{1}H	^{13}C	^{1}H	^{13}C	^{1}H	^{13}C	^{1}H	^{13}C
Pulse program (Bruker)	t1ir	t1irig	t1ir	t1irig	t1ir	t1irig	t1ir	t1irig
90° pulse width $p1$ (μs)	13.3	11.0	14.0	10.0	11.5	12.5	10.0	15.0
acquisition time aq (s)	0.27	0.50	0.10	0.40	0.30	0.10	0.2	0.1
Preacquisition delay de (μs)	4.5	15.5	4.5	18.0	6.5	18.0	10.0	18.0
Number of scans ns	16	3k– 30k	16	256 – 2k	16	1k – 2k	8	64 – 128

Table S2. Acquisition parameters for ^{1}H and ^{13}C and ^{31}P NMR experiments at room temperature (1.4 – 9.4 T).

Spectrometer	Spinsolve 60	DRX 200			AVII 400		
Probe type		TBO			BBFO		
Nucleus	^{1}H	^{1}H	^{13}C	^{31}P	^{1}H	^{13}C	^{31}P
90° pulse width $p1$ (μs)	10.6	13.3	11.0	7.8	14.0	10.0	12.0
Used pulse width	10.6	4.4	7.3	7.8	1.4	6.7	12.0
acquisition time aq (s)	0.65	0.44	0.43	0.03	0.50	0.16	0.02
Relaxation delay $d1$ (s)	1.0	0.05	0.2	0	0.05	0.20	0.02
Preacq. delay de (μs)	20	6.0	20.0	6.0	6.5	6.5	4.5
Number of scans ns	16	1k	65k	8k	512	46k	4k

Table S3. Acquisition parameters for ^{1}H and ^{13}C and ^{31}P NMR experiments at room temperature (14.1 – 18.8 T).

Spectrometer	AVIII 600					AVIII 800	
Probe type	BBO			CP-QNP		CP-TCI	
Nucleus	^{1}H	^{13}C	^{31}P	^{1}H	^{13}C	^{1}H	^{13}C
90° pulse width $p1$ (μs)	11.5	8.1	12.0	13.5	12.0	10.0	15.0
Used pulse width	1.2	5.4	12.0	1.4	8.0	1.0	5.0
acquisition time *aq* (s)	0.40	0.20	0.03	0.1	0.16	0.2	0.16
Relaxation delay $d1$ (s)	1.0	0.05	0.0	1.0	0.05	1.0	0.5
Preacq. delay *de* (μs)	6.5	6.5	6.0	10.0	18.0	10.0	18.0
Number of scans *ns*	128	20k	16k	128	4k	8	1k

Table S4. R_1 relaxation rates of selected nuclei of complex **1** at various external fields in Hz.

B (T)	18.8	14.1	9.4	4.7	1.4
C^6	88.5	62.1	34.1	10.9	
C^1	52.9	33.9	17.8	5.2	
H^2	48.5	34.2	18.8	6.2	1.6
C^{11}	43.7	26.3	13.4	6.1	
H^4	28.6	19.6	10.8	3.5	1.2
C^5	21.2	13.7	6.3	2.6	
C^2	9.5	7.2	5.2	2.3	
C^4	8.5	5.8	3.8	2.3	
C^3	8.3	5.2	3.1	2.8	
C^8	3.0	1.9	1.3	1.2	
C^7	1.7	0.6	0.3	0.2	

Table S5. Chemical shifts (600.1 MHz, Tol-d^8) of the ^{1}H NMR resonances of complex **1** at various temperatures.

T (K)	H^4	H^2	H^8	H^{11}	H^9	H^{12}
217.93	25.41	7.36	4.81	-9.61	-95.4	-4783
223.13	25.01	7.41	4.73	-9.35	-93.3	-4676
228.35	24.63	7.45	4.66	-9.13	-91.0	-4572
233.13	24.27	7.49	4.59	-8.92	-89.0	-4474
238.91	23.92	7.52	4.53	-8.70	-86.3	-4385
244.09	23.60	7.56	4.47	-8.51	-84.2	-4289
249.34	23.29	7.59	4.41	-8.33	-82.1	-4199
254.59	22.99	7.62	4.35	-8.15	-80.4	-4114
259.89	22.71	7.64	4.30	-7.99	-78.7	-4032
265.16	22.44	7.67	4.24	-7.83	-76.9	-3952
270.48	22.19	7.69	4.19	-7.68	-75.3	-3877
275.78	21.94	7.71	4.14	-7.54	-73.7	-3803
281.05	21.71	7.73	4.10	-7.40	-72.2	-3733
286.38	21.49	7.74	4.05	-7.27	-70.8	-3667
291.75	21.28	7.76	4.01	-7.14	-69.4	-3598
297.17	21.05	7.78	3.97	-7.00	-67.9	-3529
302.77	20.88	7.79	3.93	-6.89	-66.8	-3475
307.90	20.69	7.80	3.89	-6.78	-65.5	-3415
313.07	20.51	7.81	3.86	-6.67	-64.4	-3356
318.30	20.34	7.82	3.82	-6.57	-63.2	-3301
323.51	20.17	7.83	3.79	-6.47	-62.1	-3247
328.68	20.01	7.84	3.76	-6.37	-61.1	-3195
333.84	19.86	7.85	3.73	-6.28	-60.0	-3144
338.97	19.71	7.86	3.69	-6.19	-59.0	-3088
344.22	19.57	7.87	3.67	-6.10	-58.1	-3048

Table S6. Chemical shifts (150.9 MHz, Tol-d^8) of the ^{13}C NMR resonances of complex **1** at various temperatures.

T (K)	C^3	C^1	C^5	C^{11}	C^8	C^2	C^4	C^7	C^6	C^{10}	C^9
217.93	270.3	208.9	203.6	n.o.	74.0	32.4	8.4	-2.8	-226.7	-570.2	-1312.7
223.13	267.4	207.1	202.1	n.o.	73.1	34.4	10.8	-2.0	-217.7	-556.6	-1280.7
228.35	264.6	205.4	200.7	n.o.	72.3	36.2	13.2	-1.2	-209.3	-543.2	-1251.2
233.13	261.9	203.8	199.3	n.o.	71.5	38.1	15.5	-0.4	-201.1	-530.3	-1222.6
238.91	259.3	202.2	198.0	n.o.	70.7	39.8	17.7	0.4	-193.2	-517.7	-1194.7
244.09	256.8	200.7	196.8	n.o.	69.9	41.5	19.9	1.1	-185.3	-505.4	-1167.5
249.34	254.5	199.3	195.7	n.o.	69.2	43.1	21.9	1.8	-177.9	-494.0	-1141.3
254.59	252.2	197.9	194.6	118.1	68.5	44.7	23.9	2.5	-170.7	-483.0	-1117.1
259.89	250.0	196.6	193.6	117.4	67.8	46.2	25.8	3.1	-163.8	-472.3	-1093.7
265.16	247.9	195.3	192.6	115.8	67.2	47.7	27.7	3.8	-157.2	-462.1	-1071.0
270.48	245.9	194.1	191.7	114.3	66.5	49.1	29.4	4.4	-150.7	-452.4	-1049.2
275.78	243.9	193.0	190.8	112.2	65.9	50.4	31.1	4.9	-144.5	-443.0	-1028.0
281.05	242.1	191.8	190.0	111.0	65.4	51.7	32.8	5.5	-138.6	-434.1	-1008.2
286.38	240.3	190.8	189.2	109.2	64.8	52.9	34.4	6.0	-132.8	-425.3	-988.2
291.75	238.6	189.7	188.5	108.1	64.3	54.2	35.9	6.6	-127.2	-417.0	-969.7
297.17	236.8	188.7	187.7	107.0	63.7	55.4	37.5	7.1	-121.5	-408.6	-951.0
302.77	235.3	187.7	187.1	105.3	63.3	56.5	38.9	7.6	-116.5	-401.1	-934.4
307.90	233.8	186.8	186.5	104.2	62.8	57.6	40.3	8.0	-111.4	-393.6	-916.8
313.07	232.3	185.9	185.9	102.9	62.3	58.6	41.6	8.5	-106.5	-386.3	-900.7
318.30	230.8	185.0	185.3	101.8	61.9	59.7	42.9	9.0	-101.8	-379.2	-885.0
323.51	229.5	184.2	184.7	100.6	61.4	60.7	44.2	9.4	-97.2	-372.5	-870.6
328.68	228.1	183.4	184.2	99.4	61.0	61.7	45.5	9.8	-92.7	-365.9	-856.3
333.84	226.8	182.6	183.7	98.5	60.6	62.6	46.7	10.2	-88.3	-359.4	-841.3
338.97	225.5	181.8	183.2	97.4	60.2	63.5	47.8	10.6	-84.0	-353.3	-827.8
344.22	224.3	181.1	182.7	96.4	59.8	64.4	49.0	11.0	-79.8	-347.3	-814.0

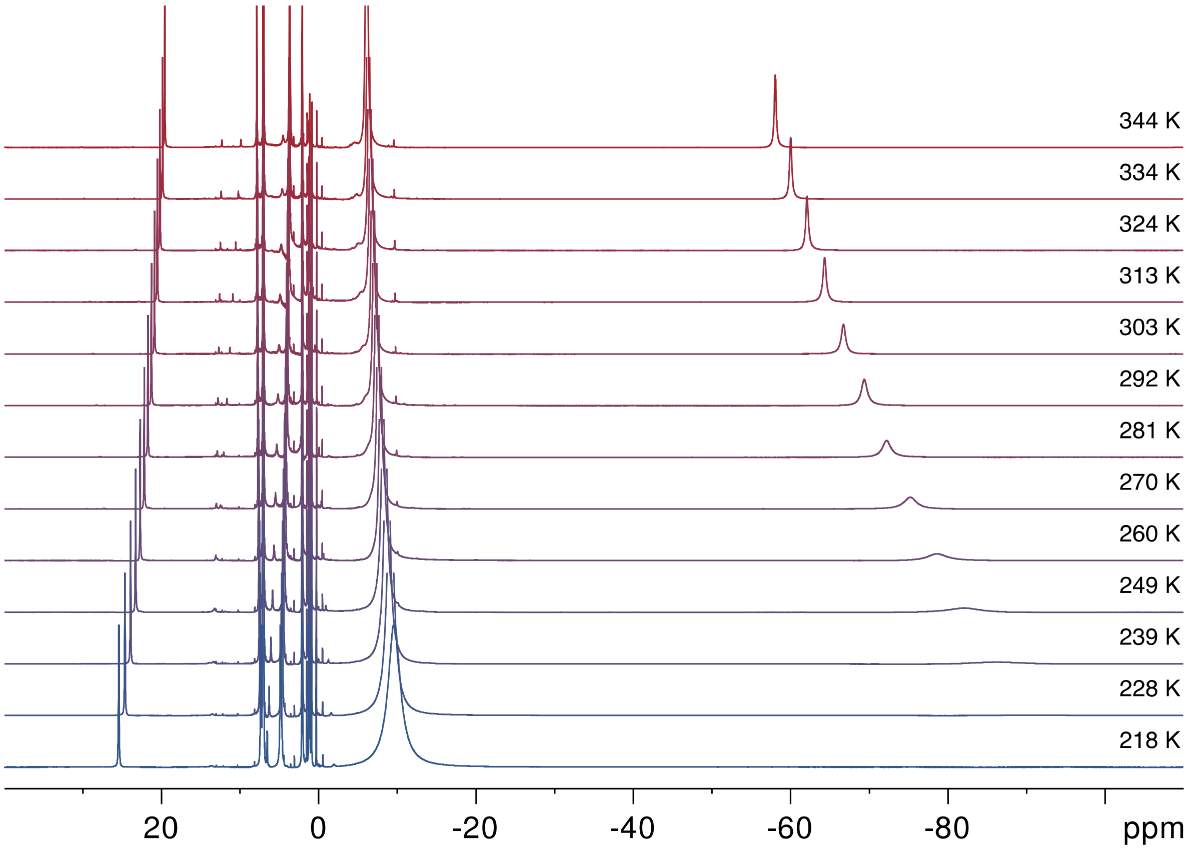

Figure S1. ^{1}H NMR spectra (600.1 MHz, Tol-d^8) of complex **1** at various temperatures.

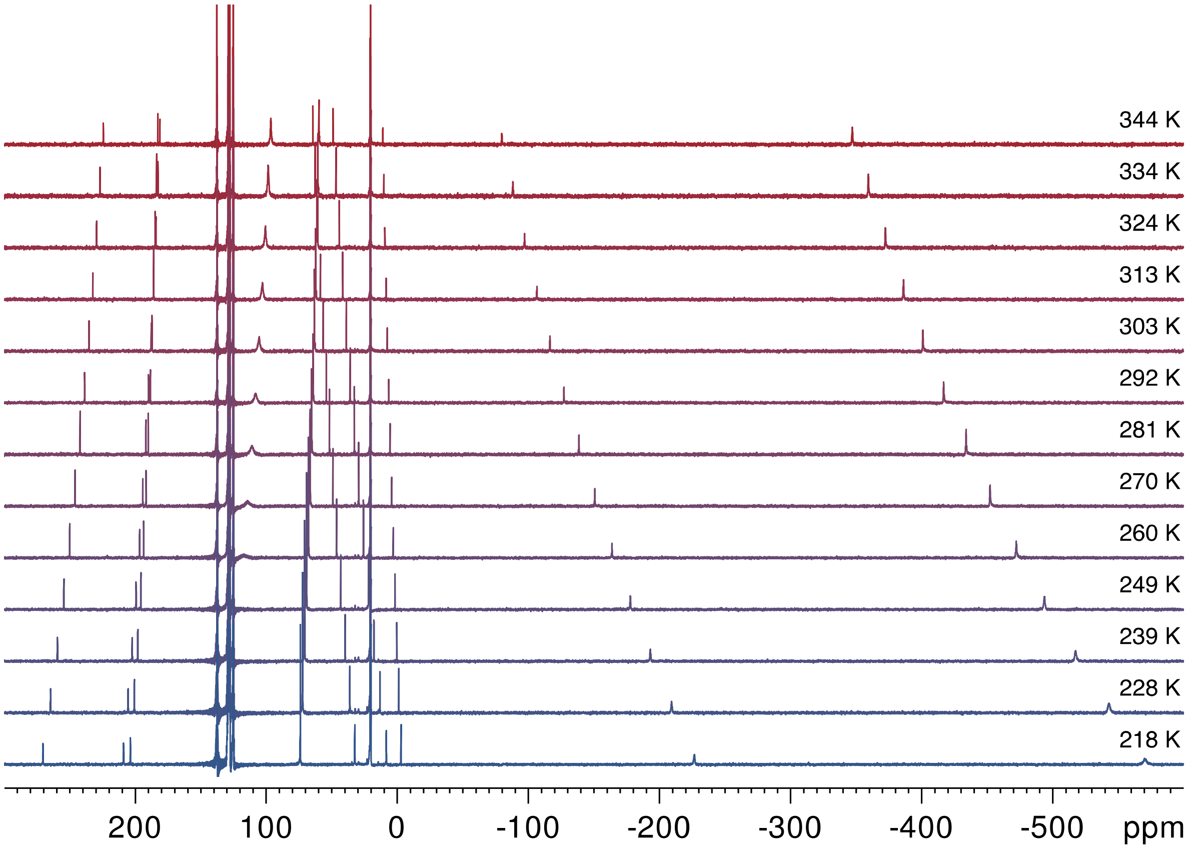

Figure S2. ^{13}C NMR spectra (150.9 MHz, Tol-d^8) of complex **1** at various temperatures.

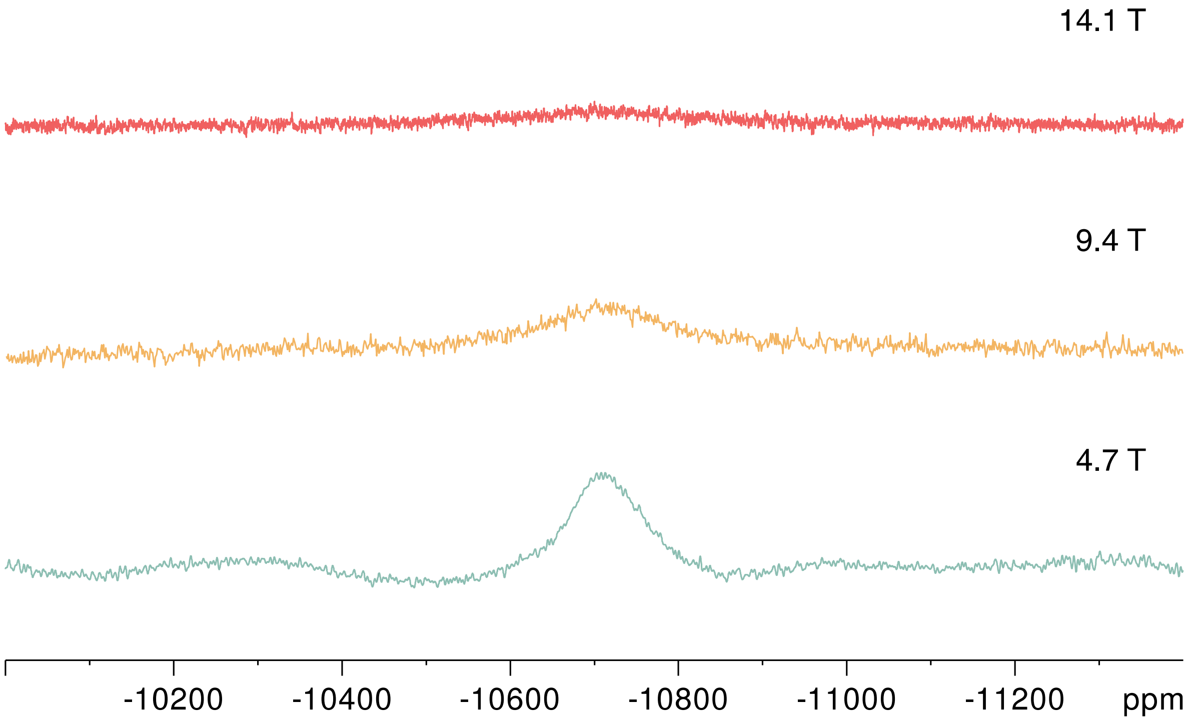

Figure S3. ^{31}P NMR spectra (295 K, Tol-d^8) of complex **1** at various external fields.

S3 FD-FT THz-EPR Spectroscopy

FD-FT THz-EPR was performed at the THz beamline of the synchrotron BESSY II, Helmholtz-Zentrum Berlin, Germany.[6] This spectrometer allows for THz-EPR measurements from ~ 0.1 to 11 THz (~ 3 – 370 cm^{-1}) employing a fully evacuated quasi-optical beam path, a high-resolution FTIR spectrometer (IFS 125, Bruker), a superconducting high field magnet (Oxford Spectromag 4000, B_0 = +11 T - 11T) with variable temperature insert (T = 1.5 K – 300 K) and liquid He cooled bolometer detectors. Herein, the internal source of the FTIR spectrometer (Hg arc lamp) was used as THz source in combination with a pumped liquid He cooled (1.5 K) bolometer. This set-up is most sensitive in the frequency range between 17 and 45 cm^{-1}. A larger spectral window, yet significantly lower detection sensitivity, is achieved between ~ 20 and 370 cm^{-1} employing a bolometer operated at 4.2 K. A detailed description of the spectrometer can be found elsewhere.[7,8] Samples were measured as pellets (15.9 mg powder sample of **1** and 117.8 mg Polyethylene), pressed inside a glove box, vacuum-sealed and immediately frozen in liquid nitrogen. SH simulations were performed with EasySpin[9] and its extension for frequency-domain EPR.[10]

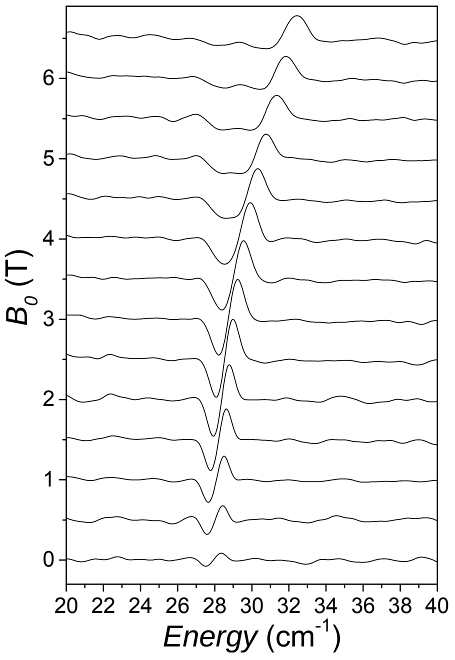

Figure S4. Magnetic Division Spectra (MDS) FD-FT THz-EPR spectra of pressed powder pellets of **1** measured at a temperature of 5 K. The MDS for the magnetic field B_0 is obtained by dividing the raw spectrum measured at B_0 by the spectrum measured at B_0 + 1 T. Data is shown offset for B_0 and rescaled with a global normalization factor. In MDS, EPR resonances appear as negative (EPR resonance at B_0) and positive (EPR resonance at B_0 + 1 T) deviations from **1**.

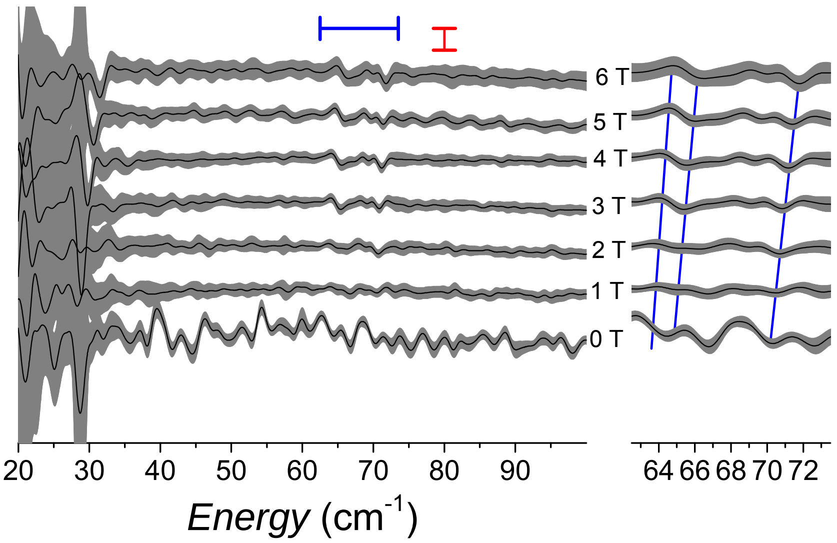

Figure S5. Magnetic Division Spectra (MDS) FD-FT THz-EPR spectra of pressed powder pellets of **1** measured at a temperature of 5 K. The gray shaded area marks the uncertainty obtained from the standard deviation of the recorded spectra. The red bar denotes 1%. The blue bar in the second plot marks the region that is shown magnified on the right side. Blue lines are guide to the eye to follow the magnetic field dependent spectral features.

Figure S4 depicts field dependent FD-FT THz-EPR spectra measured with a 1.5 K Si bolometer. At zero magnetic field a single EPR resonance at 27.5 cm^{-1} is observed. Upon application of an external magnetic field, this resonance shifts to higher excitation energies. Notably, the field dependence is clearly nonlinear. In addition, FD-FT THz EPR spectra were recorded with the broadband spectrometer configuration employing a Hg arc lamp as THz source and a bolometer cooled to 4.2 K. In the spectra depicted in Figure S5 three field-dependent features have been observed between 64 and 70 cm^{-1}. However, due to the much lower detection sensitivity in this frequency range, these features are much less pronounced. A direct comparison of the transitions depicted in Figure S4 and Figure S5 is difficult due to the differences in the detection sensitivity of the two detection modes.

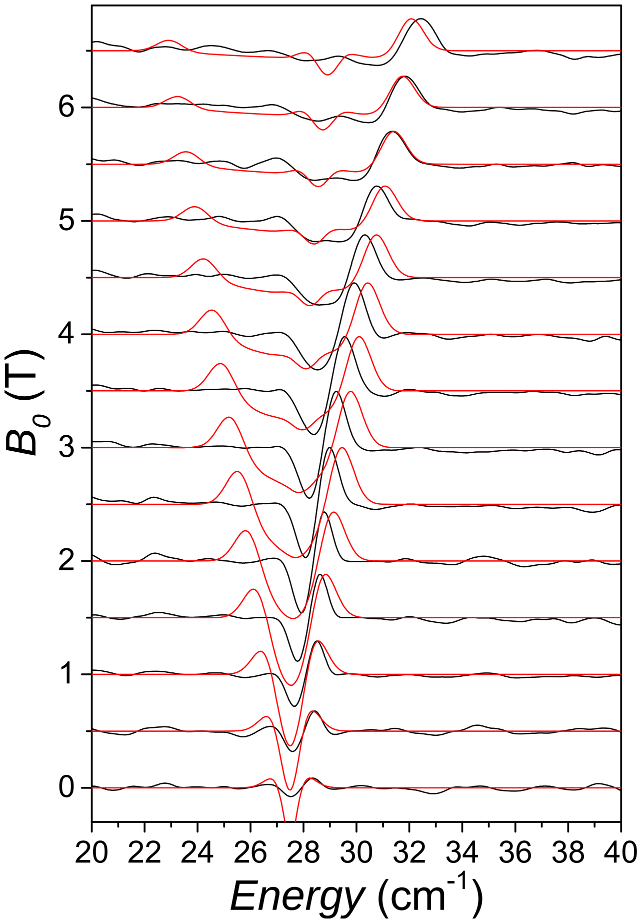

Figure S6. Experimental (black, same data as in Figure S1) and simulated (red) FD-FT THz-EPR magnetic division spectra (MDS) of pressed powder samples of **1** measured at T = 5 K. Simulations with Eq 8[4] were obtained with SH parameters: D = 27.5 cm^{-1}, E = 0, and an isotropic g = 1.4. The field dependence of the high-energy line remain linear even for anisotropic g values (simulations not shown).

The simplest interpretation for the resonance at 27.5 cm^{-1} for S = 1 is an axial ZFS with D = 27.5 cm^{-1} and vanishing E. However, simulations of the field dependent spectra with this parameter set deviate significantly from the experimental spectra (see Figure S6). First, the simulations do not reproduce the relative intensities observed with and without field, Furthermore, the position of the high-energy maximum increases linearly with increasing magnetic field in the simulation, which was not observed experimentally. Therefore, we discarded axial ZFS from the further considerations.

S4 SQUID Magnetometry

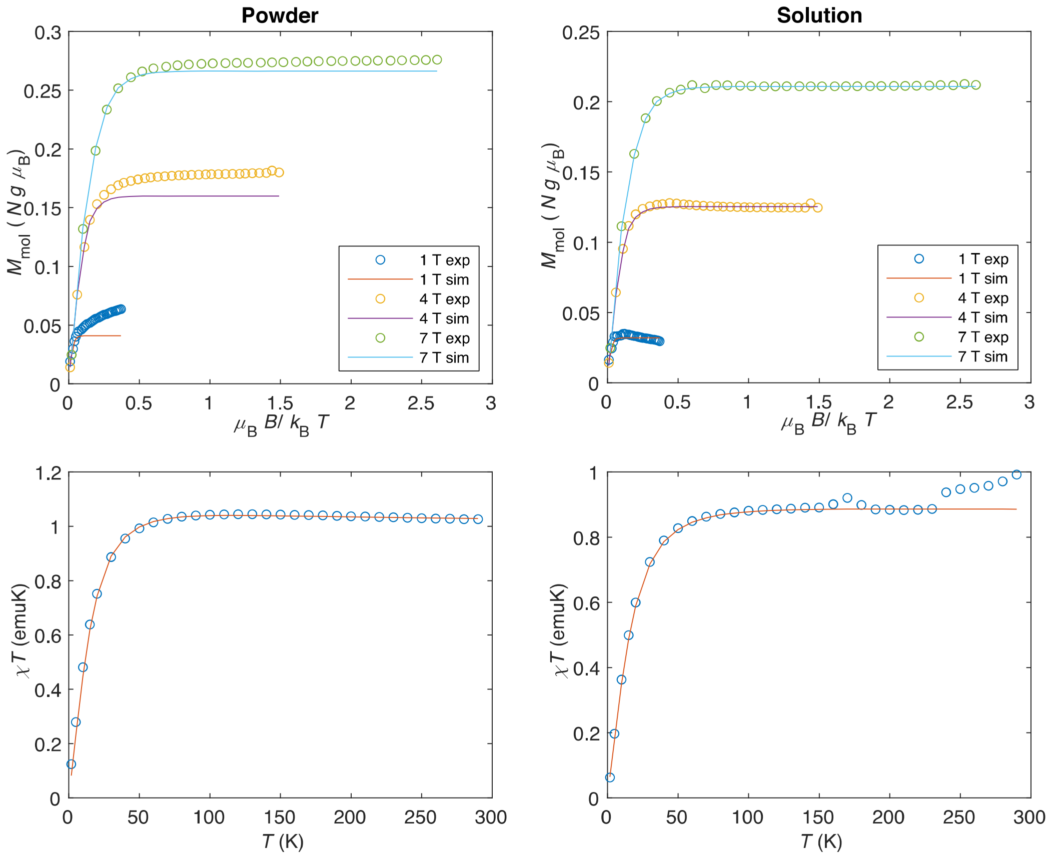

Figure S7. Experimental (circles) and simulated (solid lines) reduced magnetisation at 1 T, 4 T and 7 T of complex **1** as powder (top left) and in toluene solution (top right) with a concentration of 9.67 × 10^{-2} mol/l. Magnetic susceptibility is shown as χT product vs. temperature for powder (bottom left) and solution (bottom right). Simulations were obtained with D = –54 cm^{-1}, $|E|$ = 14 cm^{-1}, ($|E/D|$ = 0.26), $g_{\perp}$ = 1.72, $g_{\parallel}$ = 2.49 and a TIP of 0.0015 emu, for the powder samples and D = –54 cm^{-1}, $|E|$ = 14 cm^{-1}, ($|E/D|$ = 0.26), g_x = 1.40, g_y = 1.90, g_z = 2.23 and a TIP of 0.00059 emu for the solution samples.

S5 NMR Shift Analysis

Table S7. Atomic coordinates (in Å) of DFT (B3LYP/6-311G(d,p) + def2-TZVP(Fe only)) S = 1 optimized symmetrized molecular structure, components of hyperfine tensor computed with TPSSH/def2-TZVP with dipolar part normalized per electron (ppm/Å^3) and diamagnetic shielding calculated for S = 0 with M06-L/6-31G(d) in ppm not adjusted for reference (189.6621 ppm for carbon and 32.1833 ppm for hydrogen). Both hyperfine and shielding tensors are symmetric and only unique components are shown.

Atom	X	Y	Z	A_{xx}	A_{xy}	A_{xz}	A_{yy}	A_{yz}	A_{zz}	σ_{xx}	σ_{xy}	σ_{xz}	σ_{yy}	σ_{yz}	σ_{zz}
Fe	0	0	0	1521048	0	–14934	615903	0	2286466	–14126	0	5379	–48808	0	–14000
P	0.21545	2.27554	0.08741	–123751	–5276	–918	–155188	–2238	–124037	182	–12	29	211	25	165
P	0.21545	–2.27554	0.08741	–123751	5276	–918	–155188	2238	–124037	182	12	29	211	–25	165
N	–2.01928	0	–0.23098	–40185	0	–4534	–55238	0	–34669	–72	0	–97	186	0	208
C	–2.77544	–1.10878	–0.63527	–1638	2729	837	–7628	88	–8690	30	–28	–60	7	–3	97
C	–2.56495	–2.48524	–0.41042	1443	2366	–994	760	619	349	56	–7	–75	18	9	111
C	–3.4809	–3.38823	–0.95139	–977	962	615	–760	310	–2141	90	10	–39	–14	17	120
C	–4.61526	–3.01363	–1.6984	1465	526	–1360	–20	259	2304	37	–20	–79	1	–1	111
C	–4.83392	–1.65101	–1.8799	–699	455	993	–1273	107	–2250	42	6	–82	57	11	122
C	–3.93864	–0.7186	–1.35237	1707	522	–948	–2160	350	–14	53	–1	–61	–8	3	122
C	–3.93864	0.7186	–1.35237	1707	–522	–948	–2160	–350	–14	53	1	–61	–8	–3	122
C	–4.83392	1.65101	–1.8799	–699	–455	993	–1273	–107	–2250	42	–6	–82	57	–11	122
C	–4.61526	3.01363	–1.6984	1465	–526	–1360	–20	–259	2304	37	20	–79	1	1	111
C	–3.4809	3.38823	–0.95139	–977	–962	615	–760	–310	–2141	90	–10	–39	–14	–17	120
C	–2.56495	2.48524	–0.41042	1443	–2366	–994	760	–619	349	56	7	–75	18	–9	111
C	–2.77544	1.10878	–0.63527	–1638	–2729	837	–7628	–88	–8690	30	28	–60	7	3	97
C	–1.45033	3.01426	0.45334	–16935	–710	369	–11076	161	–15267	162	15	13	124	–2	168
C	–1.45033	–3.01426	0.45334	–16935	710	369	–11076	–161	–15267	162	–15	13	124	2	168
C	–5.5765	–4.07112	–2.27313	–374	280	130	–533	118	–656	143	4	–1	150	2	141
C	–5.10992	–5.4979	–1.9203	35	245	79	123	92	–145	146	–11	5	167	–8	142
C	–6.99417	–3.85935	–1.69292	1136	199	56	774	40	695	172	–4	–14	141	4	140
C	–5.6531	–3.96469	–3.80983	859	186	211	724	137	850	133	3	4	140	–2	178
C	–5.5765	4.07112	–2.27313	–374	–280	130	–533	–118	–656	143	–4	–1	150	–2	141
C	–5.6531	3.96469	–3.80983	859	–186	211	724	–137	850	133	–3	4	140	2	178
C	–6.99417	3.85935	–1.69292	1136	–199	56	774	–40	695	172	4	–14	141	–4	140
C	–5.10992	5.4979	–1.9203	35	–245	79	123	–92	–145	146	11	5	167	8	142
C	1.25694	2.90266	1.5762	–6895	1261	67	–3569	1209	–7282	144	–2	4	120	–6	150
C	0.51742	2.43218	2.84568	981	593	469	3864	2367	3197	148	9	–15	138	–21	164
C	2.65597	2.26203	1.58582	1505	1979	1115	632	778	–45	168	–23	–1	146	0	144
C	1.39787	4.43437	1.63482	–2986	412	164	–2205	417	–2929	142	0	–1	164	–6	148
C	0.79072	3.07297	–1.55837	–7843	772	–1	–4246	–990	–8398	142	–3	–5	112	3	148
C	–0.28186	2.72238	–2.60889	–670	–291	151	2314	–2180	1116	163	12	17	134	20	161
C	2.1201	2.45246	–2.02115	784	1757	–1202	1608	–1361	462	166	–22	–7	142	11	142
C	0.9293	4.60339	–1.48825	–3106	252	–90	–2230	–401	–2977	145	1	3	162	8	149
C	1.25694	–2.90266	1.5762	–6895	–1261	67	–3569	–1209	–7282	144	2	4	120	6	150
C	0.51742	–2.43218	2.84568	981	–593	469	3864	–2367	3197	148	–9	–15	138	21	164
C	2.65597	–2.26203	1.58582	1505	–1979	1115	632	–778	–45	168	23	–1	146	0	144
C	1.39787	–4.43437	1.63482	–2986	–412	164	–2205	–417	–2929	142	0	–1	164	6	148
C	0.79072	–3.07297	–1.55837	–7843	–772	–1	–4246	990	–8398	142	3	–5	112	–3	148
C	–0.28186	–2.72238	–2.60889	–670	291	151	2314	2180	1116	163	–12	17	134	–20	161
C	2.1201	–2.45246	–2.02115	784	–1757	–1202	1608	1361	462	166	22	–7	142	–11	142
C	0.9293	–4.60339	–1.48825	–3106	–252	–90	–2230	401	–2977	145	–1	3	162	–8	149
H	1.57026	0	0	–26015	0	–97	–91134	0	–83045	–111	0	9	245	0	86
H	–3.30318	–4.44077	–0.7616	24	639	95	488	143	–376	30	1	5	19	1	23
H	–5.70112	–1.29706	–2.42497	453	214	335	–209	91	–191	22	1	2	34	1	19
H	–5.70112	1.29706	–2.42497	453	–214	335	–209	–91	–191	22	–1	2	34	–1	19
H	–3.30318	4.44077	–0.7616	24	–639	95	488	–143	–376	30	–1	5	19	–1	23
H	–1.64364	2.73015	1.49155	138	–1985	–731	2609	1767	–190	29	13	4	11	–11	34

H	−1.43308	4.10448	0.4183	−3318	−800	6	−1172	261	−3339	32	3	2	17	−1	27
H	−1.43308	−4.10448	0.4183	−3318	800	6	−1172	−261	−3339	32	−3	2	17	1	27
H	−1.64364	−2.73015	1.49155	138	1985	−731	2609	−1767	−190	29	−13	4	11	11	34
H	−5.80116	−6.23359	−2.33991	15	164	58	62	66	−104	30	2	2	35	1	28
H	−5.07334	−5.65628	−0.84	32	260	34	136	36	−178	29	0	2	30	−4	31
H	−4.1164	−5.70234	−2.32727	−37	241	93	175	141	−151	33	−4	1	30	1	27
H	−7.68919	−4.60272	−2.09688	185	141	60	46	40	−23	35	3	1	30	0	27
H	−7.37887	−2.86725	−1.94132	227	138	101	−105	39	−143	33	−3	0	34	0	25
H	−6.99514	−3.95327	−0.60398	189	214	15	−59	14	−159	32	0	−3	29	−1	33
H	−6.34002	−4.71785	−4.20533	114	136	116	46	91	25	29	2	4	30	2	33
H	−4.67444	−4.12973	−4.26807	5	194	186	−31	191	−3	31	−1	−2	28	1	34
H	−6.01423	−2.98468	−4.12849	108	145	211	−108	96	−29	27	−2	3	33	−3	32
H	−6.34002	4.71785	−4.20533	114	−136	116	46	−91	25	29	−2	4	30	−2	33
H	−6.01423	2.98468	−4.12849	108	−145	211	−108	−96	−29	27	2	3	33	3	32
H	−4.67444	4.12973	−4.26807	5	−194	186	−31	−191	−3	31	1	−2	28	−1	34
H	−7.68919	4.60272	−2.09688	185	−141	60	46	−40	−23	35	−3	1	30	0	27
H	−6.99514	3.95327	−0.60398	189	−214	15	−59	−14	−159	32	0	−3	29	1	33
H	−7.37887	2.86725	−1.94132	227	−138	101	−105	−39	−143	33	3	0	34	0	25
H	−5.80116	6.23359	−2.33991	15	−164	58	62	−66	−104	30	−2	2	35	−1	28
H	−4.1164	5.70234	−2.32727	−37	−241	93	175	−141	−151	33	4	1	30	−1	27
H	−5.07334	5.65628	−0.84	32	−260	34	136	−36	−178	29	0	2	30	4	31
H	0.38221	1.34891	2.85878	−2658	530	918	−732	3521	3574	33	1	−1	46	−20	23
H	−0.4574	2.89943	2.95812	−1103	−292	−215	632	1691	308	35	1	0	22	−9	31
H	1.1086	2.69966	3.72165	−561	352	469	131	1088	663	30	−1	1	29	−5	36
H	2.60366	1.17663	1.51112	2568	2265	2376	−1607	905	−1332	25	−13	−3	48	−4	30
H	3.15175	2.51671	2.52481	91	838	726	−270	601	−346	34	−4	3	30	−2	33
H	3.28573	2.62549	0.77826	788	1399	358	127	283	−787	33	−8	−3	26	−2	33
H	1.96169	4.83461	0.79132	−270	487	84	804	211	−439	27	1	0	22	−3	34
H	1.94005	4.70724	2.54617	−577	363	156	147	436	−514	30	1	2	28	1	32
H	0.43171	4.94021	1.67127	−486	152	25	932	414	−318	31	−3	1	25	−1	29
H	−1.24576	3.16841	−2.38185	−758	−764	444	958	−1424	−128	42	3	−4	18	5	29
H	−0.42162	1.64235	−2.70311	−2463	−616	863	−71	−3665	2611	39	4	−2	37	19	24
H	0.04322	3.09931	−3.58082	−683	21	−54	370	−1161	540	32	0	0	27	4	36
H	2.95864	2.742	−1.39344	421	1370	−592	207	−565	−691	34	−8	2	25	4	31
H	2.33409	2.80235	−3.0336	−233	708	−676	39	−847	79	32	−4	−3	28	4	33
H	2.07293	1.36254	−2.04384	836	2367	−2999	−1019	−1918	2	23	−15	5	45	12	23
H	−0.00816	5.0863	−1.20662	−501	46	26	970	−313	−408	32	−2	−1	24	1	28
H	1.20475	4.98264	−2.4785	−880	235	−92	6	−449	−781	29	0	−2	28	−1	32
H	1.70628	4.92303	−0.79188	−331	422	−85	849	−211	−442	28	1	3	22	2	33
H	−0.4574	−2.89943	2.95812	−1103	292	−215	632	−1691	308	35	−1	0	22	9	31
H	0.38221	−1.34891	2.85878	−2658	−530	918	−732	−3521	3574	33	−1	−1	46	20	23
H	1.1086	−2.69966	3.72165	−561	−352	469	131	−1088	663	30	1	1	29	5	36
H	2.60366	−1.17663	1.51112	2568	−2265	2376	−1607	−905	−1332	25	13	−3	48	4	30
H	3.28573	−2.62549	0.77826	788	−1399	358	127	−283	−787	33	8	−3	26	2	33
H	3.15175	−2.51671	2.52481	91	−838	726	−270	−601	−346	34	4	3	30	2	33
H	0.43171	−4.94021	1.67127	−486	−152	25	932	−414	−318	31	3	1	25	1	29
H	1.94005	−4.70724	2.54617	−577	−363	156	147	−436	−514	30	−1	2	28	−1	32
H	1.96169	−4.83461	0.79132	−270	−487	84	804	−211	−439	27	−1	0	22	3	34
H	−0.42162	−1.64235	−2.70311	−2463	616	863	−71	3665	2611	39	−4	−2	37	−19	24
H	−1.24576	−3.16841	−2.38185	−758	764	444	958	1424	−128	42	−3	−4	18	−5	29
H	0.04322	−3.09931	−3.58082	−683	−21	−54	370	1161	540	32	0	0	27	−4	36
H	2.07293	−1.36254	−2.04384	836	−2367	−2999	−1019	1918	2	23	15	5	45	−12	23
H	2.33409	−2.80235	−3.0336	−233	−708	−676	39	847	79	32	4	−3	28	−4	33
H	2.95864	−2.742	−1.39344	421	−1370	−592	207	565	−691	34	8	2	25	−4	31
H	1.70628	−4.92303	−0.79188	−331	−422	−85	849	211	−442	28	−1	3	22	−2	33
H	1.20475	−4.98264	−2.4785	−880	−235	−92	6	449	−781	29	0	−2	28	1	32
H	−0.00816	−5.0863	−1.20662	−501	−46	26	970	313	−408	32	2	−1	24	−1	28

Table S8. Fitting parameters of pNMR signals obtained with different computational methods.

		χ_{iso}, Å^3	$\Delta\chi_{xx}$, Å^3	$\Delta\chi_{yy}$, Å^3	χ_{xz}, Å^3	**RMSE**	**Error degree of freedom**
**B3LYP/def2-TZVP**	all signals	0.069(1)	0.040(3)	-0.03(1)	-0.01±0.03	36	14
	selected	0.069(1)	0.040(3)	-0.03(1)	-0.02(1)	6.38	6
	selected	0.068(1)	0.036(4)	-0.04(1)	fixed at 0	10.5	7
**TPSSH/def2-TZVP**	selected	0.063(1)	0.046(3)	-0.04(1)	0.0003±0.006	6.35	6
	selected	**0.063(1)**	**0.046(2)**	**-0.04(1)**	**fixed at 0**	**6.07**	**7**
**B3LYP/EPR-II**	selected	0.069(1)	0.040(3)	-0.03(1)	-0.007±0.007	6.37	6
	selected	0.069(1)	0.037(2)	-0.03(1)	fixed at 0	6.15	7
**BP86/def2-TZVP**	selected	0.069(2)	0.08(1)	-0.02±0.02	-0.006±0.02	17.8	6
	selected	0.068(1)	0.08(1)	-0.03(1)	fixed at 0	16.7	7
**HF/def2-TZVP**	selected	could not reach a reasonable agreement with experiment				186	6

Table S9. Ligand field parameters calculated with CASSCF(6,5)/NEVPT2/def2-TZVP for 5 quintet, 45 triplet and 50 singlet states for X-Ray structure of the complex.

```
------------------------------
AILFT MATRIX ELEMENTS (NEVPT2)
------------------------------

E0              =  0.018882711 a.u. =     0.514 eV =    4144.3 cm**-1
H(dxy   ,dxy   )=  0.022269585 a.u. =     0.606 eV =    4887.6 cm**-1
H(dyz   ,dxy   )= -0.008951501 a.u. =    -0.244 eV =   -1964.6 cm**-1
H(dyz   ,dyz   )= -0.009372948 a.u. =    -0.255 eV =   -2057.1 cm**-1
H(dz2   ,dxy   )=  0.010316368 a.u. =     0.281 eV =    2264.2 cm**-1
H(dz2   ,dyz   )= -0.002174074 a.u. =    -0.059 eV =    -477.2 cm**-1
H(dz2   ,dz2   )=  0.005891424 a.u. =     0.160 eV =    1293.0 cm**-1
H(dxz   ,dxy   )= -0.026264992 a.u. =    -0.715 eV =   -5764.5 cm**-1
H(dxz   ,dyz   )=  0.008126089 a.u. =     0.221 eV =    1783.5 cm**-1
H(dxz   ,dz2   )= -0.016922601 a.u. =    -0.460 eV =   -3714.1 cm**-1
H(dxz   ,dxz   )=  0.029199490 a.u. =     0.795 eV =    6408.5 cm**-1
H(dx2-y2,dxy   )=  0.043447367 a.u. =     1.182 eV =    9535.6 cm**-1
H(dx2-y2,dyz   )= -0.004922911 a.u. =    -0.134 eV =   -1080.5 cm**-1
H(dx2-y2,dz2   )=  0.018012799 a.u. =     0.490 eV =    3953.4 cm**-1
H(dx2-y2,dxz   )= -0.047085588 a.u. =    -1.281 eV =  -10334.1 cm**-1
H(dx2-y2,dx2-y2)=  0.065308713 a.u. =     1.777 eV =   14333.6 cm**-1
B               =  0.004634455 a.u. =     0.126 eV =    1017.1 cm**-1
C               =  0.014493589 a.u. =     0.394 eV =    3181.0 cm**-1  (C/B=  3.13)

The ligand field one electron eigenfunctions:
Orbital    Energy (eV)  Energy(cm-1)      dxy        dyz        dz2        dxz       dx2-y2
    1          0.000         0.0         0.385827   0.817483  -0.068328  -0.284001  -0.312304
    2          0.271      2187.2         0.235910   0.235612   0.642989   0.671387   0.156967
    3          0.341      2753.1         0.667697  -0.517675   0.107807  -0.033941  -0.522894
    4          0.499      4028.0        -0.398108  -0.030229   0.725561  -0.474701  -0.298022
    5          3.925     31653.9         0.437234  -0.085480   0.209371  -0.492027   0.718048
```

S6 Relaxation Theory

This work deals with the rare case of NMR spectroscopy of a nucleus so close to the unpaired electron that (a) the point dipole approximation breaks down; (b) the contact part of the hyperfine coupling cannot be ignored. The system is also far outside the Zeeman limit in which most spin relaxation theories are formulated. Before we deal with the matter numerically, it is useful to review pre-existing – and now inapplicable – analytical results.

Solomon-Bloembergen-Morgan (SBM),[11–13] and Gueron ("Curie")[14] mechanisms assume an effective electron magnetic moment μ_{eff} (from the isotropic part of the susceptibility tensor) and use point-dipole approximation for the hyperfine coupling:[15]

$$\begin{aligned}
R_1^{\text{dip}} &= \frac{2}{15}\left(\frac{\mu_0}{4\pi}\right)^2 \frac{\gamma_{\text{N}}^2 \mu_{\text{eff}}^2}{r^6}\left[3j\left(\omega_{\text{N}},\tau_{\text{R},T_{1\text{E}}}\right)+6j\left(\omega_{\text{E}}+\omega_{\text{N}},\tau_{\text{R},T_{2\text{E}}}\right)+j\left(\omega_{\text{E}}-\omega_{\text{N}},\tau_{\text{R},T_{2\text{E}}}\right)\right] \\
R_2^{\text{dip}} &= \frac{1}{15}\left(\frac{\mu_0}{4\pi}\right)^2 \frac{\gamma_{\text{N}}^2 \mu_{\text{eff}}^2}{r^6}\left[\begin{array}{l} 4j\left(0,\tau_{\text{R},T_{1\text{E}}}\right)+3j\left(\omega_{\text{N}},\tau_{\text{R},T_{1\text{E}}}\right)+6j\left(\omega_{\text{E}},\tau_{\text{R},T_{2\text{E}}}\right) \\ +6j\left(\omega_{\text{E}}+\omega_{\text{N}},\tau_{\text{R},T_{2\text{E}}}\right)+j\left(\omega_{\text{E}}-\omega_{\text{N}},\tau_{\text{R},T_{2\text{E}}}\right) \end{array}\right] \\
R_1^{\text{Curie}} &= \frac{6}{5}\left(\frac{\mu_0}{4\pi}\right)^2 \frac{\omega_{\text{N}}^2 \mu_{\text{eff}}^4}{(3kT)^2 r^6} j\left(\omega_{\text{N}},\tau_{\text{R}}\right) \\
R_2^{\text{Curie}} &= \frac{1}{5}\left(\frac{\mu_0}{4\pi}\right)^2 \frac{\omega_{\text{N}}^2 \mu_{\text{eff}}^4}{(3kT)^2 r^6}\left[4j\left(0,\tau_{\text{R}}\right)+3j\left(\omega_{\text{N}},\tau_{\text{R}}\right)\right]
\end{aligned} \tag{S1}$$

where the spectral power density and the characteristic times are:

$$j(\omega,\tau)=\frac{\tau}{1+\omega^2\tau^2}, \qquad \tau_{\text{R},T_{1\text{E}}}=\left(\frac{1}{\tau_{\text{R}}}+\frac{1}{T_{1\text{E}}}\right)^{-1}, \qquad \tau_{\text{R},T_{2\text{E}}}=\left(\frac{1}{\tau_{\text{R}}}+\frac{1}{T_{2\text{E}}}\right)^{-1} \tag{S2}$$

Here, μ_0 is vacuum permeability, γ_{N} is the magnetogyric ratio of the nucleus, ω_{N} is the nuclear Zeeman frequency, ω_{E} is the electron Zeeman frequency, μ_{eff} is the effective magnetic moment of the electron, r is the electron-nuclear distance, τ_{R} is the second rank rotational correlation time of what is often assumed to be isotropic rotational diffusion, $T_{1\text{E}}$ is the longitudinal electron relaxation time, and $T_{2\text{E}}$ is the transverse electron relaxation time.

When the unpaired electrons are delocalised and the nucleus is nearby, a point electron dipole is not a good approximation – the proper hyperfine tensor must be used instead:

$$\begin{aligned} R_1^{\mathrm{HF}} &= \frac{\Delta_{\mathbf{A}}^2 S(S+1)}{90}\left[3j\left(\omega_{\mathrm{N}},\tau_{\mathrm{R},T_{1\mathrm{E}}}\right)+6j\left(\omega_{\mathrm{E}}+\omega_{\mathrm{N}},\tau_{\mathrm{R},T_{2\mathrm{E}}}\right)+j\left(\omega_{\mathrm{E}}-\omega_{\mathrm{N}},\tau_{\mathrm{R},T_{2\mathrm{E}}}\right)\right] \\ R_2^{\mathrm{HF}} &= \frac{\Delta_{\mathbf{A}}^2 S(S+1)}{180}\left[\begin{array}{l} 4j\left(0,\tau_{\mathrm{R},T_{1\mathrm{E}}}\right)+3j\left(\omega_{\mathrm{N}},\tau_{\mathrm{R},T_{1\mathrm{E}}}\right)+6j\left(\omega_{\mathrm{E}},\tau_{\mathrm{R},T_{2\mathrm{E}}}\right) \\ +6j\left(\omega_{\mathrm{E}}+\omega_{\mathrm{N}},\tau_{\mathrm{R},T_{2\mathrm{E}}}\right)+j\left(\omega_{\mathrm{E}}-\omega_{\mathrm{N}},\tau_{\mathrm{R},T_{2\mathrm{E}}}\right) \end{array}\right] \end{aligned} \quad \text{(S3)}$$

where $\Delta_{\mathbf{A}}^2$ is the second rank invariant of the hyperfine tensor:

$$\begin{aligned} \Delta_{\mathbf{A}}^2 &= a_{\mathrm{XX}}^2 + a_{\mathrm{YY}}^2 + a_{\mathrm{ZZ}}^2 - a_{\mathrm{XX}}a_{\mathrm{YY}} - a_{\mathrm{XX}}a_{\mathrm{ZZ}} - a_{\mathrm{YY}}a_{\mathrm{ZZ}} \\ &+ \frac{3}{4}\left[\left(a_{\mathrm{XY}}+a_{\mathrm{YX}}\right)^2+\left(a_{\mathrm{XZ}}+a_{\mathrm{ZX}}\right)^2+\left(a_{\mathrm{YZ}}+a_{\mathrm{ZY}}\right)^2\right] \end{aligned} \quad \text{(S4)}$$

Eq (S1) is adequate at distances greater than 10 Å from the metal, and Eq (S3) is applicable at any distance in the Zeeman limit.

When the contact component of the hyperfine coupling is significant, an additional relaxation mechanism turns up: rapid electron relaxation creates stochastic noise in the isotropic hyperfine coupling operator, the resulting nuclear relaxation mechanism is called scalar relaxation of the second kind. It depends on the isotropic hyperfine coupling constant A_{iso}:[16]

$$\begin{aligned} R_1^{\mathrm{SRSK}} &= \frac{2A_{\mathrm{iso}}^2 S(S+1)}{3} j\left(\omega_{\mathrm{E}}-\omega_{\mathrm{N}},T_{2\mathrm{e}}\right) \\ R_2^{\mathrm{SRSK}} &= \frac{A_{\mathrm{iso}}^2 S(S+1)}{3}\left[j\left(0,T_{1\mathrm{e}}\right)+j\left(\omega_{\mathrm{E}}-\omega_{\mathrm{N}},T_{2\mathrm{e}}\right)\right] \end{aligned} \quad \text{(S5)}$$

in this case, the role of correlation times is played by electron relaxation times.

More subtle effects appear in systems with significant susceptibility anisotropy.[17,18] For example, Curie mechanism pops an extra term due to the antisymmetry in the paramagnetic shielding tensor which arises from the combination of susceptibility anisotropy and hyperfine coupling anisotropy:[19,20]

$$R_1^{\text{Curie+CSA}} = \frac{1}{2}\Lambda_{\boldsymbol{\sigma}}^2\omega_{\text{N}}^2\frac{\tau_{\text{R}}}{1+9\omega_{\text{N}}^2\tau_{\text{R}}^2} + \frac{2}{15}\Delta_{\boldsymbol{\sigma}}^2\omega_{\text{N}}^2\frac{\tau_{\text{R}}}{1+\omega_{\text{N}}^2\tau_{\text{R}}^2}$$
$$R_2^{\text{Curie+CSA}} = \frac{1}{4}\Lambda_{\boldsymbol{\sigma}}^2\omega_{\text{N}}^2\frac{\tau_{\text{R}}}{1+9\omega_{\text{N}}^2\tau_{\text{R}}^2} + \frac{1}{45}\Delta_{\boldsymbol{\sigma}}^2\omega_{\text{N}}^2\left(4\tau_{\text{R}} + \frac{3\tau_{\text{R}}}{1+\omega_{\text{N}}^2\tau_{\text{R}}^2}\right) \tag{S6}$$

where $\Lambda_{\boldsymbol{\sigma}}^2$ is the first rank invariant of the total chemical shielding tensor:

$$\Lambda_{\boldsymbol{\sigma}}^2 = \left(\sigma_{\text{XY}} - \sigma_{\text{YX}}\right)^2 + \left(\sigma_{\text{XZ}} - \sigma_{\text{ZX}}\right)^2 + \left(\sigma_{\text{YZ}} - \sigma_{\text{ZY}}\right)^2 \tag{S7}$$

and the total shielding tensor is the sum of the diamagnetic and paramagnetic contributions:

$$\boldsymbol{\sigma} = \boldsymbol{\sigma}_0 - \chi\mathbf{A} \tag{S8}$$

A notable case was recently described where the negative sign in this equation resulted in a reduction of shielding anisotropy, and thus a slowdown in the nuclear relaxation.[21]

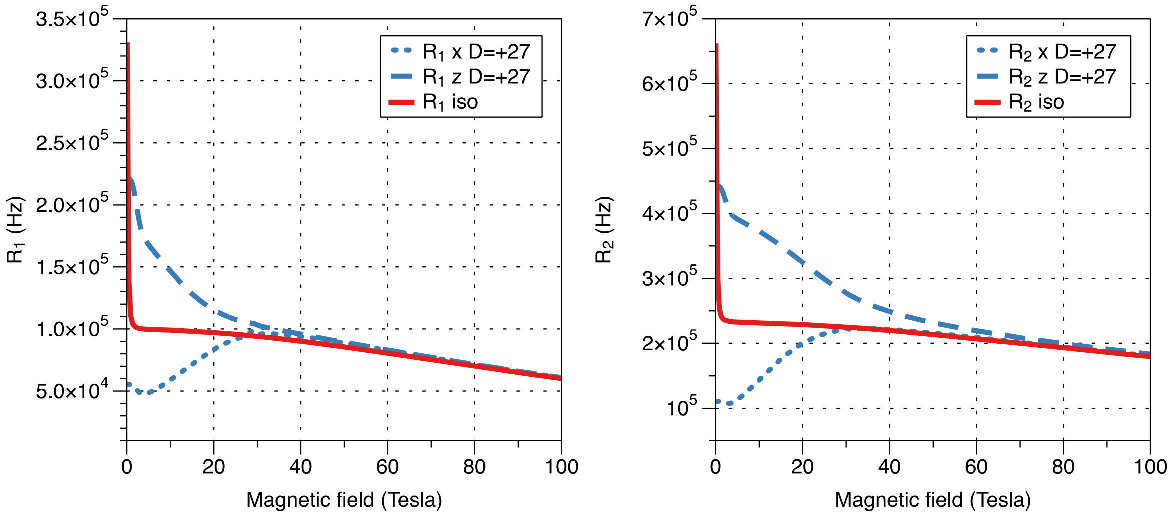

Figure S8. Simulated contact/dipolar relaxation rates (longitudinal – R_1, and transverse – R_2) as a function of magnetic field. The model represents a point paramagnetic centre with S = 1 and ^{1}H located 1.57 Å away along either along x-axis (dotted lines) or z-axis (dashed lines) and an axial ZFS with *D* = +27 cm^{-1}. The g-tensor is assumed to be isotropic with $g = 2$, and electron relaxation rate is 3.3×10^{10} s^{-1}.

S7 Literature

[1] J. C. Ott, H. Wadepohl, M. Enders, L. H. Gade, *J. Am. Chem. Soc.* **2018**, *140*, 17413–17417.

[2] M. J. Frisch, G. W. Trucks, H. B. Schlegel, G. E. Scuseria, M. A. Robb, J. R. Cheeseman, G. Scalmani, V. Barone, G. A. Petersson, H. Nakatsuji, et al., **2016**.

[3] Y. Zhao, D. G. Truhlar, *J. Phys. Chem. A* **2008**, *112*, 6794–6799.

[4] a) F. Neese, *WIREs Comput. Mol. Sci.* **2012**, *2*, 73–78. b) F. Neese, *WIREs Comput. Mol. Sci.* **2018**, *8*, 4–9.

[5] A. G. Webb, in *Annu. Rep. NMR Spectrosc.*, Academic Press, **2002**, pp. 1-67.

[6] K. Holldack, A. Schnegg, *J. large-scale Res. Facil. JLSRF* **2016**, *2*, A51.

[7] A. Schnegg, J. Behrends, K. Lips, R. Bittl, K. Holldack, *Phys. Chem. Chem. Phys.* **2009**, *11*, 6820.

[8] J. Nehrkorn, K. Holldack, R. Bittl, A. Schnegg, *J. Magn. Reson.* **2017**, *280*, 10–19.

[9] S. Stoll, A. Schweiger, *J. Magn. Reson.* **2006**, *178*, 42–55.

[10] J. Nehrkorn, J. Telser, K. Holldack, S. Stoll, A. Schnegg, *J. Phys. Chem. B* **2015**, *119*, 13816–13824.

[11] I. Solomon, *Phys. Rev.* **1955**, *99*, 559–565.

[12] N. Bloembergen, *J. Chem. Phys.* **1957**, *27*, 572–573.

[13] N. Bloembergen, L. O. Morgan, *J. Chem. Phys.* **1961**, *34*, 842–850.

[14] M. Gueron, *J. Magn. Reson.* **1975**, *19*, 58–66.

[15] I. Bertini, C. Luchinat, G. Parigi, Eds. , *Solution NMR of Paramagnetic Molecules: Applications to Metallobiomolecules and Models*, Elsevier, New York, **2016**.

[16] A. Abragam, *The Principles of Nuclear Magnetism*, Oxford University Press, London, **1961**.

[17] E. A. Suturina, I. Kuprov, *Phys. Chem. Chem. Phys.* **2016**, *18*, 26412–26422.

[18] L. Lang, E. Ravera, G. Parigi, C. Luchinat, F. Neese, *J. Phys. Chem. Lett.* **2020**, *11*, 8735–8744.

[19] E. A. Suturina, K. Mason, C. F. G. C. Geraldes, N. F. Chilton, D. Parker, I. Kuprov, *Phys. Chem. Chem. Phys.* **2018**, *20*, 17676–17686.

[20] A. J. Vega, D. Fiat, *Mol. Phys.* **1976**, *31*, 347–355.

[21] H. W. Orton, I. Kuprov, C.-T. Loh, G. Otting, *J. Phys. Chem. Lett.* **2016**, *7*, 4815–4818.
